# Maternal depressive symptomatology and its association with early child development in socioeconomically deprived Mexican households

**DOI:** 10.1007/s44192-026-00477-6

**Published:** 2026-05-19

**Authors:** Amado D. Quezada-Sánchez, Miia S. Vesterinen, Tarja I. Kinnunen, Carmen Hernández-Chávez, Angélica García-Martínez, Abby Madrigal-Ramírez, Raquel García-Feregrino, Evelyn Fuentes-Rivera, Armando García-Guerra, Edson Serván-Mori

**Affiliations:** 1 Unit of Health Sciences, Faculty of Social Sciences, Tampere University, Tampere, Finland; 2 Center for Evaluation and Survey Research, National Institute of Public Health, Cuernavaca, Morelos México; 3 Department of Developmental Neurobiology, National Institute of Perinatology Isidro Espinosa de los Reyes, Mexico City, Mexico; 4 Lucy Family Institute for Data and Society, University of Notre Dame, Notre Dame, IN USA; 5 Center for Health Systems Research, National Institute of Public Health, Cuernavaca, Morelos Mexico; 6 Center for Demographic, Urban and Environmental Studies, College of Mexico A.C, Mexico City, Mexico; 7 Center for Nutrition and Health Research, National Institute of Public Health, Cuernavaca, Morelos Mexico

**Keywords:** Early childhood development, Neurodevelopment, Bayley-III, Undernutrition, Maternal depression, Indigenous

## Abstract

We performed a cross-sectional analysis of 1060 mother-child dyads to study the association between maternal depressive symptomatology and child development among children aged 0 to 38 months from 24 communities of Oaxaca Mexico. Maternal depressive symptomatology was measured with the Center for Epidemiological Studies depression scale (CES-D). Child outcomes included WHO nutritional status indicators, a development evaluation test, and Bayley-III scales. We fitted logistic regressions for binary and linear regressions for continuous outcomes with multiple sociodemographic covariates. Children whose mothers had clinically significant depressive symptomatology (CES-D ≥ 16) had higher odds of stunting (covariate-adjusted odds ratio (AOR) = 1.32 95% confidence interval (CI): 1.04, 1.69) and lower socioemotional Bayley-III composite scores. Clinically significant maternal depressive symptomatology was associated with lower odds of normal neurodevelopment in male children (AOR = 0.60 95%CI: 0.41, 0.88) but not in females. Maternal clinically significant depressive symptomatology was associated with lower means of the Bayley-III language scores among children from Indigenous language speaking households. Public health interventions are needed to prevent maternal depression and suboptimal care practices among the most vulnerable.

*Trial registration* ClinicalTrials.gov ID NCT04210362. Study registration date 2019,07,14.

**Supplementary Information:**

The online version contains supplementary material available at 10.1007/s44192-026-00477-6.

## Introduction

Depression is a mood disorder that limits daily functioning; it can manifest with different combinations of symptoms, including feelings of worthlessness, loss of energy, changes in sleep or appetite, and lowered ability to think or concentrate [34, 35]. Women are especially vulnerable to depression and its associated health effects during postpartum [24]. A review of postpartum depression prevalence based on data from 28 low- and middle-income countries, documented a median of 19% (interquartile interval:13.2%,28.4%) across countries, these prevalence are generally higher compared to estimates from high income countries [40]. In Mexico, the prevalence of maternal depressive symptomatology was 19.9% in 2012 (95%CI: 18.2%,21.7%) [12].

Maternal depression has detrimental consequences for the well-being of mothers and their rapidly developing young children. Mothers with depression experience a lowered functional capacity and struggle more to provide adequate care practices, such as breastfeeding, stimulation, and sensitive interactions [16, 22, 32, 36]. This results in a higher risk of suboptimal child physical and neurological development with long-lasting effects [45].

Evidence from 11 low and middle income countries has shown that maternal depression is associated with undernutrition [52]. In regard to associations between maternal depression and early childhood neurodevelopment, international evidence has been synthetized in three metanalyses [17, 33, 48]. The results from Liu and cols. (2017) were based on 14 published studies (1997–2013), 13 of them from high income countries. The authors found that preschool children whose mothers had high depressive symptoms exhibited a lower mean of cognitive development scores compared to children of mothers with low depressive symptoms (Cohen’s d = − 0.25, 95%CI: − 0.39, − 0.12). The meta-analysis by Fan and cols. (2024) examined different dimensions of neurodevelopment and their associations with perinatal depression among children aged < 2 years using data with longitudinal designs from 23 studies. Most studies were from high- and middle-income countries. The authors reported significant associations for the cognitive (Cohen’s d = − 0.19, 95%CI: − 0.30, − 0.08, 13 studies), language (Cohen’s d = − 0.24, 95%CI: − 0.40, − 0.07; 13 studies) and motor dimensions (Cohen’s d = − 0.15, 95%CI: − 0.26, -0.05; 14 studies) and a significant association for the socioemotional dimension among studies using Bayley-III Scales (Cohen’s d = − 0.18, 95%CI: − 0.29, -0.06; 3 studies), these studies were from high income countries. On the other hand, the meta-analysis by Rogers and cols. (2020) found a statistically significant association between postnatal maternal depression and socioemotional problems for children aged < 2 years based on 5 studies from high income countries (correlation = 0.26, 95%CI: 0.17, 0.35).

In Mexico, few studies have focused on the relationship between maternal depression and early child development. A study in rural areas found that having higher maternal depressive symptoms was associated with more behavior problems in young children [19]. A study using nationally representative data from 2012 found that among mothers with low socioeconomic status, maternal depressive symptomatology was related to a higher risk of not having breastfed their children, but no significant association was found with child undernutrition [13]. To the best of our knowledge, there are no published studies in Mexico addressing this relationship in socially deprived populations or in areas with a high percentage of Indigenous population. International data have shown that Indigenous groups are characterized by poorer health and social outcomes compared to non-Indigenous groups [2]. The negative consequences of maternal depression on early childhood development can be exacerbated for socially deprived groups, given the presence of stressors from occupational status, violence, and lack of access to health services [13, 37].

We aimed to evaluate the association between maternal depressive symptomatology and child development outcomes and nutritional status among young children from Oaxaca, one of the States with the highest social and economic marginalization and with the highest proportion of Indigenous population in Mexico (Instituto Nacional de Estadística y Geografía, [31]). Results from the present study will shed light on the depressive symptomatology status of vulnerable mothers and its association with child development while contributing to fill the gap in the literature for Indigenous groups that may share commonalities across Latin America. This is important since much of the research has been performed in high income societies and its conclusions are not directly transferable to these contexts. It is now recognized that cognitive and motivational processes are heterogeneous across the different world populations [27].

## Methodology

### Study design and participants

This was a cross-sectional observational study. We analyzed baseline data from a study that was initially designed to assess the effect of an educational intervention on early childhood development in the state of Oaxaca, Mexico [43]. The intervention was designed by “Un Kilo de Ayuda, A.C.,” a non-governmental organization. Due to the COVID-19 health emergency, the original study was suspended prematurely in March 2020. Potential participants were purposively contacted (1) through meetings organized by local authorities and (2) home visits after reviewing local population registries [43, 51]. Baseline data were obtained from 1179 households in 24 communities from July 2019 to March 2020 in the State of Oaxaca in Mexico. Data was collected electronically using the Research Electronic Data Capture (REDCaP) application during the household visits [26]. The children’s ages ranged from 0 to 38 months. For this study, we considered only caregiver-child dyads in which the primary caregiver was mother of the child (*n* = 1073). We then excluded 2 observations with missing or inconsistent maternal age and 1 observation with out-of-range child age. After an analysis of missing data, we identified the set of analysis variables with less than 0.5% of missing values and specified them as predictors in our multiple imputation by chained equations (MICE) [54]. This resulted in a sample size of 1060 dyads (99.0%) for analysis using MICE and a sample size of 886 dyads (82.6%) for complete case analysis. More details on the MICE procedure are provided at the end of the methods section.

### Depressive symptomatology

Depressive symptomatology was assessed with the Center for Epidemiologic Studies Depression Scale (CES-D) [44]. The CES-D has been validated in multiple population groups [39, 53]. A study performed in women from a highly marginalized rural zone in the *Mixteca* (an Indigenous group in Mexico) region assessed the psychometric properties of the CES-D scale and concluded that it showed adequate reliability and validity [7].

Trained interviewers applied the CES-D questionnaire, which is comprised of 20 items about the frequency of depressive symptoms during the last week; it was coded as 0 for “less than a day,” 1 for “1–2 days,” 2 for “3–4 days,” and 3 for “5–7 days.” An overall score is obtained by summing the coded responses for the 20 symptoms, with the score ranging from 0 to 60. The CES-D can be categorized to detect the presence of clinically significant depressive symptomatology. A meta-analysis of 22 studies showed that a cutoff of 16 reached 87% (95% CI: 82, 91) sensitivity and 70% (95% CI: 65, 75) specificity using standardized diagnostic interviews based on the international classification of diseases or the Diagnostic and Statistical Manual of Mental Disorders classification system as a gold standard [53]. We used this cutoff to define a binary depressive symptoms exposure variable.

### Outcome variables

Anthropometric measurements were obtained by trained and standardized personnel [25] using SECA 872™ digital scales and SECA 206™ portable stadiometers. For simplicity, we use the term height to refer to length (measured in a recumbent position for children aged < 24 months) or stature (measured as standing height for children aged ≥ 24 months). Nutritional status was determined after calculating Z scores using the 2006 WHO child growth standards (World Health Organization Multicentre Growth Reference Study Group, [55]). Stunting was defined as height for age Z< -2, underweight as weight for age Z<-2, low weight for height as weight for height Z< -2, and overweight (including obesity) as weight for height Z > 2.

Early childhood development was assessed with the Child Development Evaluation Test 2nd Edition or “Evaluación del Desarrollo Infantil” (EDI-II) in Spanish (Comisión Nacional de Protección Social en Salud. Secretaría de Salud, [10]). This test was designed in Mexico for the early detection of neurodevelopmental issues across 14 age groups among children aged 1–60 months. It was validated using the Battelle Developmental Inventory 2nd edition as a gold standard; it reached 81% sensitivity (95% confidence intervals CI: 75%, 86%) and 61% specificity (95% CI:54%, 67%) [47]. Through direct observation, the test evaluates five developmental skill areas: gross motor, fine motor, language, social, and cognitive. The first four areas apply to all children, whereas the cognitive area applies to children 37 months or older. Apart from these areas, the test performs a neurological exploration, identifies biological risk factors, and detects warning and alarm signs of delayed child development. Results are coded into a traffic-light scale for each area. Except for a few age-group-specific differences, these categories can be summarized as: GREEN Normal development— the activities for the corresponding age group were successfully performed (2 out of 2 items for ages ≤12 months or 2 out of 3 items for older children); YELLOW Developmental lag – the activities for the corresponding age group were not successfully performed but those from the preceding age group were succesfully completed; RED At risk of developmental delay— The activities for the corresponding and preceding age group were not successfully performed. The development areas along with the other aspects of the test are considered to obtain a result. Children with a RED result were referred to a specialist for further evaluation.

Early childhood development was also measured with the Bayley Scales of Infant and Toddler Development 3rd edition (BSID-III) [5]. A subsample of 530 children was selected from the pool of recruited children with baseline nutritional status measurements and whose caregivers were their mothers. Selection was performed randomly within 10 strata defined by intervention status and five categories of the propensity to participate in the intervention. The BSID-III is considered the reference standard for developmental assessment and can be applied to children aged 1 to 42 months [4]. It includes cognitive, language, and motor domains assessed through observation and socioemotional behavior assessed through parental reporting. Composite scores based on a comparison to a normative age-matched sample are calculated for the cognitive, language and motor scales. A score of 100 is interpreted as mid-average functioning; a score below 85 (< reference mean—1 standard deviation (SD)) indicates mild impairment or at risk of developmental delay; and a score below 70 (< reference mean – 2 SD) is interpreted as moderate/severe impairment [14]. Subtest scales include receptive language, expressive language, fine motor and gross motor subdomains; these are expressed as scaled scores ranging from 1 to 19. Three trained psychologists applied the development tests in the field. Inter-rater agreement was assessed by applying the BSID-III instrument to 30 children, they achieved an inter-rater reliability coefficient of 0.90.

### Other explanatory variables and potential confounders

Data on sociodemographic and general characteristics were collected through interviewer-administered questionnaires by trained field personnel. Child characteristics included age in months, sex, and whether there was a < 5-year-old sibling living in the household. Maternal characteristics included age in years categorized into three levels (15–19, 20–29, and 30–49), completed education level (none or elementary, middle school, high school, and college or university), type of health insurance (none, “Seguro Popular”, social security), occupational status (having a job, housework, housework with extra activities, self-employed and other), marital status (single, free union, married, and separated/divorced/widowed) and parity (number of times has given birth to a live neonate).

Household characteristics included a wealth index, reported monthly income from all sources, Indigenous status, and number of social programs the household is affiliated with. The wealth index was calculated by conducting principal component analysis. We extracted the first principal component from a set of variables that included: type of household materials (floor, walls, roof), services (gas stove, water facilities, toilet) and possession of goods (automobile, television or computer, electric appliances, etc.). The resulting index absorbed 27% of the total variance. Variable weights and their correlation with the first principal component are shown in Supplementary Material Table ST1. The wealth index was categorized into three levels (low, medium, high) through tertiles. Indigenous status was classified into three categories based on the responses of the parents (“Non-Indigenous”, “Indigenous without Indigenous language spoken” if any of the parents self-identified as Indigenous according to their culture and none of them spoke an Indigenous language, and “Indigenous with Indigenous language spoken” if any of the parents self-identified as Indigenous according to their culture and any of them spoke an Indigenous language). The categories of household monthly income in the questionnaire were “less than 3000”, “3000 to 5000”, “5001 to 7000,” and “more than 7000” Mexican pesos. We converted the income bracket limits to euros using the 2020 purchase power parity exchange rates for private consumption (OECD, [38]). The number of social programs from which the household received benefits was categorized into three levels (none, one, two to four).

### Statistical analyses

We described the distribution of general and sociodemographic characteristics, as well as child development outcomes by maternal depressive symptomatology status and in the overall sample. Means and SDs were obtained for continuous type variables and percentages for categorical variables. Homogeneity tests based on overall tests of significance from multinomial logistic regression were performed to assess whether the distributions of each categorial sociodemographic characteristic varied with maternal CES-D categories; for children’s age we compared means between maternal CES-D categories using linear regression. We also described the distribution of sociodemographic characteristics by sex and by household Indigenous status.

Logistic regression models were fitted for binary outcomes and linear regression for continuous-type outcomes with two specifications for the linear predictor: (1) the CES-D score without other covariates, (2) the CES-D score plus all general and sociodemographic characteristics as described in the variables section. We standardized the CES-D scores with the overall mean and SD before fitting our models. Associations between maternal depressive symptomatology and binary outcomes are presented as crude odds ratios (OR) and covariate-adjusted odds ratios (AOR) for specifications 1 and 2, respectively. Associations between maternal depressive symptomatology and continuous-type outcomes are presented as mean differences (MD) and covariate-adjusted mean differences (AMD), these differences correspond to contrasts between CES-D exposure levels that differ by 1 SD. Although strictly speaking the CES-D score is a discrete variable, we interpret it as continuous throughout. We also fitted our models using maternal depressive symptomatology as categorical exposure. We did not apply our models to wasting since its prevalence was low and it was not present in some covariate categories.

All standard errors were adjusted for data dependencies within communities [49]. All model coefficients are presented with their 95% CI. The level of statistical significance was set at $alpha\:=0.05$. For continuous outcomes and the two-category maternal depressive symptomatology exposure, mean differences were expressed in pooled standard deviation units (Cohens’ d) and reported when statistically significant [9].

We explored whether sex and Indigenous status moderated the associations between maternal depressive symptomatology and early child development, by adding the interactions between each one of these potential moderators and the CES-D score to specification 2, in separated models. We also performed these analyses with the categorical depressive symptomatology exposure.

As a sensitivity analysis, we refitted our models with a fractional power monotonic transformation of the CES-D score to produce an exposure variable with a more symmetrical distribution. We also performed augmented inverse probability weighting analyses and estimated the average treatment effect on the treated (ATT) for comparing the mean of the group whose mothers had clinically significant depressive symptomatology (CES-D ≥ 16) with its estimated counterfactual based on observed characteristics [8]. We used the same set of covariates in our specification 2 as predictors of the outcomes and depressive symptomatology status. For the BSID-III analyses by household Indigenous status, maternal age and schooling were specified as continuous since maternal symptomatology status did not vary in some categories of these characteristics in one of the Indigenous status groups.

### Handling of missing data

We used 10 predictors and imputed values for seven variables using MICE [54]. The predictors included child’s characteristics (age as continuous, sex, and whether there was a < 5-year-old sibling living in the household), maternal characteristics (age as continuous, occupational status, marital status, completed education level, and type of health insurance), and household characteristics (Indigenous status and affiliation to social programs). We performed MICE with a total of m = 20 repetitions. The seven variables with imputed values were household income (9.1%), maternal depressive symptomatology (5.3%), parity (4.1%), EDI-II result (3.7%), stunting (3.6%), low weight for height (3.6%), overweight-obesity (3.6%), and wealth index category (2.3%). In addition, we imputed missing data for the anthropometric variables shown in the descriptive analyses (3.6%). More details on the imputation procedure are provided in the Supplementary Material (Tables ST2-ST3). We refitted our models without multiple imputations (complete case analysis).

All analyses were performed in Stata MP (StataCorp. 2025. Stata Statistical Software: Release 19. College Station, TX: StataCorp LLC).

## Results

The analysis sample consisted of 1060 mother-child dyads, 886 (83.6%) had complete data on all covariates, the CES-D score, and any of our outcomes. An analysis of missing data and a brief description of the MICE procedure is presented in Supplementary Material Tables ST2-ST3. Child age ranged from 0 to 38 months (mean = 19.6, SD = 9.0) and 46.5% were female (Table 1). Regarding maternal characteristics, 9.0% were adolescents (15–19 years), 22.1% had elementary or no schooling, about 40% were self-employed or had a job and 9.0% were single. Approximately two thirds of households were Indigenous and 25% had an Indigenous language speaking parent; 79% of households had a monthly income of 314 euros or less. Clinically significant depressive symptomatology (CESD ≥ 16) was present in 27.8% of mothers, compared with other mothers, they were less likely to have completed high school or college/university, less likely to be married, and more likely to be in the low-wealth category. A histogram of the maternal CES-D scores is available in Supplementary Material Figure SF1, the distribution showed positive skew, a mean of 12.7 and SD = 10.0. Households in which an Indigenous language was spoken had the highest percentages in the lowest maternal schooling and income categories (40.9% and 53.7%, respectively, Supplementary Material ST4). Sociodemographic characteristics were similarly distributed across sex categories (not shown).

**Table 1:** Sociodemographic and general characteristics of mothers and their children aged 0 to 38 months from 24 communities in Oaxaca Mexico

Child characteristics	Overall (*n* = 1060)	CES-D < 16 (*n* = 765^a^)	CES-D ≥ 16 (*n* = 295^a^)	*P* ^b^
Female, %	46.5	45.9	48.1	0.454
Age in months, mean (SD)	19.6 (9.0)	19.6 (9.0)	19.8 (9.1)	0.759
*Age group in months, %*				
0 to 11	23.9	24.3	22.8	0.748
12 to 23	39.8	39.2	41.4	
24 to 38	36.3	36.5	35.8	
Has a sibling < 5 years of age, %	30.8	29.6	34.2	0.172
*Maternal characteristics*				
*Age group in years, %*				
15 to 19	9.0	8.1	11.1	0.499
20 to 29	57.2	57.6	56.1	
30 to 49	33.9	34.3	32.8	
*Completed education level, %*				
None or elementary	22.1	19.2	29.6	< 0.001
Middle school	43.3	42.0	46.6	
Highschool	24.5	27.5	16.8	
College/University	10.1	11.3	7.1	
*Health insurance, %*				
None	9.5	9.3	10.0	< 0.001
Seguro Popular	79.2	77.1	84.4	
Social Security	11.3	13.5	5.6	
*Occupation status, %*				
Has a job	20.3	20.3	20.2	0.615
Housework	42.5	41.9	43.9	
Housework with extra activities^c^	14.9	15.8	12.6	
Self-employed	19.2	18.8	20.4	
Other^d^	3.1	3.2	2.9	
*Marital status, %*				
Single	9.0	9.4	7.8	< 0.001
Free Union	53.5	50.5	61.2	
Married	32.7	35.9	24.6	
Separated/Divorced/Widowed	4.8	4.2	6.3	
Parity, number of times has given birth to a live neonate^e^, mean (SD)	2.07 (1.14)	2.03 (1.10)	2.15 (1.22)	0.166
*Household characteristics*				
*Wealth index category* ^*f*^ *, %*				
Low	33.3	27.6	48.1	< 0.001
Medium	33.4	36.3	26.1	
High	33.2	36.1	25.8	
*Reported monthly income* ^*g*^				
Less than 189€	43.3	40.9	49.8	0.067
189€ to 314€	35.7	36.8	32.9	
> 314€ to 440€	11.2	12.1	8.6	
More than 440€	9.8	10.2	8.7	
*Indigenous status, %*				
Non-Indigenous	34.0	32.0	39.0	0.041
Indigenous without Indigenous language spoken	41.1	43.8	34.1	
Indigenous with Indigenous language spoken	24.9	24.1	26.9	
*Number of social programs, %*				
None	71.6	71.8	71.1	0.438
One	22.7	22.1	24.4	
Two to four	5.7	6.1	4.5	

CES-D: Center for Epidemiologic Studies depression scale

^a^56 missing values were imputed using multiple imputations with chained equations (MICE), sample sizes of the two CES-D categories are average frequencies over m = 20 repetitions

^b^*P*-value from an overall test of significance using multinomial logistic regression for categorical variables or linear regression for continuous-type variables

^c^Extra activities such as helping in a business or selling a product

^d^Includes being a student or retired

^e^43 missing values were imputed using MICE in each repetition

^f^24 missing values were imputed using MICE in each repetition

^g^Converted from Mexican pesos to euros using the 2020 purchase power parity exchange rates for private consumption, 96 missing values were imputed using MICE in each repetition

The average height of the children was 78 (SD = 8.8) cm, and the average weight was 10.1 (SD = 2.1) kg; 23.8% were stunted, 6.0% were underweight, 1.5% had low weight for height, and 2.9% had overweight or obesity (Table 2). Results from the EDI-II test showed that 42.3% had normal development, 46.5% had a developmental lag, and 11.3% were at risk of developmental delay. Regarding the child development assessment using the BSID-III instrument in a subsample (*n* = 554), the percentage of children with mild to severe impairment were 10.3%, 23.6% and 15.5% for the cognitive, language and motor domains, respectively.

**Table 2:** Descriptive statistics of nutritional status indicators and child development outcomes by maternal depressive symptomatology status among children aged 0 to 38 months from 24 communities in Oaxaca Mexico

	Maternal depressive symptomatology		Overall
	CES-D < 16	CES-D ≥ 16	
*Physiological development: Anthropometry and nutritional status*			
	*n*=765^a^	*n*=295^a^	*n* = 1060
Height^b^, cm	78.0 (8.8)	77.8 (9.0)	77.9 (8.8)
Height for age, Z	− 1.31 (0.95)	− 1.46 (1.01)	− 1.35 (0.97)
Stunting^c^, %	21.4	30.0	23.8
Weight, kg	10.2 (2.1)	10.1 (2.2)	10.1 (2.1)
Weight for age, Z	− 0.57 (0.97)	− 0.67 (0.96)	− 0.60 (0.97)
Underweight^d^, %	5.2	8.2	6.0
Weight for height or length, Z	0.16 (1.00)	0.13 (0.93)	0.15 (0.98)
Low weight for height^e^, %	1.4	1.8	1.5
Overweight or obesity^f^, %	3.4	1.5	2.9
*Child Development Evaluation Test 2nd Edition - EDI-II*			
	*n*=765^a^	*n*=295^a^	*n* = 1060
Normal	43.9	38.1	42.3
Developmental lag	44.3	52.0	46.5
At risk of developmental delay	11.8	9.8	11.3
*Bayley Scales of Infant and Toddler Development 3rd edition - BSID-III*			
	*n*=397^a^	*n*=157^a^	*n* = 554
Cognitive composite score	95.0 (10.7)	93.3 (10.8)	94.5 (10.7)
Mild impairment^g^, %	8.0	12.2	9.2
Moderate/severe impairment^h^, %	1.0	1.3	1.1
Language composite score	92.2 (10.7)	90.0 (11.6)	91.6 (11.0)
Mild impairment^g^, %	21.6	22.5	21.8
Moderate/severe impairment^h^, %	1.3	3.2	1.8
Receptive language subtest scaled score	8.7 (2.2)	8.3 (2.3)	8.6 (2.2)
Expressive language subtest scaled score	8.6 (2.1)	8.2 (2.2)	8.5 (2.1)
Motor composite score	94.8 (12.5)	94.1 (12.5)	94.6 (12.5)
Mild impairment^g^, %	12.1	14.5	12.8
Moderate/severe impairment^h^, %	2.8	2.5	2.7
Fine motor subtest scaled score	9.8 (2.4)	9.6 (2.5)	9.7 (2.5)
Gross motor subtest scaled score	8.5 (2.4)	8.4 (2.3)	8.4 (2.4)
Socioemotional, score	91.1 (12.1)	88.0 (14.2)	90.2 (12.8)

Estimates are means (standard deviations) or percentages

CES-D: Center for Epidemiologic Studies depression scale

^a^56 missing values were imputed using multiple imputations with chained equations (MICE), sample sizes of the two CES-D categories are average frequencies over m = 20 repetitions

^b^For simplicity, the term “height” refers to length (measured in recumbent position) or stature (standing height)

^c^Height for age Z < -2

^d^Weight for age Z < -2

^e^Weight for height Z < -2

^f^Weight for height Z > 2

^g^Defined as a composite score between 70 and < 85

^h^Defined as a composite score < 70

### Associations between maternal depressive symptomatology and child nutritional status

An increase of 1 SD in the maternal CES-D score was associated with 20% higher odds of stunting (OR = 1.20 95%CI: 1.07, 1.35), 24% higher odds of underweight (OR = 1.24 95%CI: 1.02, 1.50) and 37% lower odds of overweight or obesity (OR = 0.63 95%CI: 0.40, 0.98) (Table 3). After adjusting for multiple sociodemographic characteristics, children whose mothers had clinically significant depressive symptomatology (CES-D ≥ 16) had 32% higher covariate-adjusted odds of stunting compared to other children (AOR = 1.32 95% CI: 1.04, 1.69, Table 3). The ATT showed that children whose mothers had clinically significant depressive symptomatology had 5.1% points higher prevalence of stunting compared to a situation in which their mothers had no clinically significant depressive symptomatology (95%CI: 0.3, 9.9, Table 3).

**Table 3:** Associations between maternal depressive symptomatology and child development outcomes among children aged 0 to 38 months from 24 communities in Oaxaca Mexico

Outcome	Maternal depressive symptomatology exposure				
	CES-D + 1 SD vs. CES-D		CES-D ≥ 16 vs. CES-D < 16		
	OR (95% CI)	AOR (95%CI)	OR (95% CI)	AOR (95%CI)	AIPW ATT MD (95%CI)
*Nutritional status* ^*a*^ *, n = 1060*					
Stunting^b^	1.20** (1.07, 1.35)	1.09 (0.97, 1.22)	1.57*** (1.22, 2.03)	1.32* (1.04, 1.69)	5.10* (0.34, 9.85)
Underweight^c^	1.24* (1.02, 1.50)	1.09 (0.90, 1.32)	1.64* (1.10, 2.44)	1.28 (0.85, 1.93)	2.07 (− 0.91, 5.04)
Overweight^d^	0.63* (0.40, 0.98)	0.69 (0.47, 1.01)	0.44* (0.19, 0.98)	0.48* (0.23, 0.99)	− 1.31 (− 2.76, 0.14)
*Child Development Evaluation Test 2nd Edition – EDI-II, n = 1060*					
Normal neurodevelopment	0.90 (0.81, 1.01)	0.94 (0.82, 1.07)	0.79* (0.63, 0.99)	0.86 (0.65, 1.15)	− 3.85 (− 10.62, 2.92)
*BSID-III composite scores, n = 554*					
	MD (95% CI)	AMD (95% CI)	MD (95% CI)	AMD (95%CI)	AIPW ATT MD (95%CI)
Cognitive	− 0.76* (− 1.46, − 0.05)	− 0.10 (− 0.81, 0.60)	− 1.73* (− 3.26, − 0.20)	− 0.29 (− 2.11, 1.52)	− 0.25 (− 1.75, 1.25)
Language	− 0.69 (− 2.02, 0.64)	− 0.39 (− 1.62, 0.84)	− 2.16 (− 4.86, 0.53)	− 1.43 (− 4.15, 1.29)	− 1.82 (− 4.36, 0.72)
Motor	− 0.02 (− 0.96, 0.93)	0.58 (− 0.48, 1.65)	− 0.68 (− 3.03, 1.68)	0.80 (− 1.77, 3.37)	0.65 (− 1.59, 2.88)
Socio-emotional	− 1.64* (− 2.86, − 0.41)	− 1.20 (− 2.44, 0.04)	− 3.15* (− 5.59, − 0.70)	− 2.31* (− 4.49, − 0.13)	− 2.15* (− 4.29, − 0.005)
*BSID-III subtest scaled scores, n = 554*					
	MD (95% CI)	AMD (95% CI)	MD (95% CI)	AMD (95%CI)	AIPW ATT MD (95%CI)
Receptive language	− 0.10 (− 0.37, 0.17)	− 0.07 (− 0.32, 0.17)	− 0.36 (− 0.90, 0.18)	− 0.26 (− 0.74, 0.22)	− 0.29 (− 0.69, 0.10)
Expressive language	− 0.14 (− 0.35, 0.08)	− 0.06 (− 0.27, 0.16)	− 0.38 (− 0.83, 0.06)	− 0.22 (− 0.72, 0.27)	− 0.32 (− 0.83, 0.19)
Fine motor	− 0.04 (− 0.23, 0.15)	0.06 (− 0.15, 0.26)	− 0.15 (− 0.61, 0.31)	0.08 (− 0.42, 0.58)	0.08 (− 0.37, 0.53)
Gross motor	0.03 (− 0.13, 0.19)	0.14 (− 0.05, 0.32)	− 0.07 (− 0.49, 0.36)	0.19 (− 0.29, 0.66)	0.13 (− 0.32, 0.59)
*Impaired neurodevelopment (BSID-III composite scores < 85)*					
	OR (95% CI)	AOR (95%CI)	OR (95% CI)	AOR (95%CI)	AIPW ATT MD (95%CI)
Cognitive	1.19 (0.91, 1.57)	1.05 (0.79, 1.39)	1.57 (0.96, 2.56)	1.23 (0.66, 2.28)	1.41 (− 5.32, 8.14)
Language	1.04 (0.83, 1.31)	0.99 (0.80, 1.23)	1.16 (0.74, 1.84)	1.05 (0.66, 1.66)	2.01 (− 6.53, 10.56)
Motor	0.97 (0.75, 1.27)	0.90 (0.67, 1.20)	1.18 (0.71, 1.94)	0.97 (0.57, 1.68)	− 0.81 (− 7.40, 5.79)

*p < 0.05, **p < 0.01, ***p < 0.001

CI: Confidence interval; CES-D: Center for Epidemiologic Studies depression scale; SD: overall standard deviation of the CES-D scale (= 10.0); OR: odds ratio; AOR: covariate adjusted odds ratio; MD: mean difference; AMD: covariate-adjusted mean difference; AIPW ATT MD: Augmented inverse probability weighting average treatment effect on the treated as a mean difference, for binary outcomes it was expressed in percentage points; BSID-III: Bayley Scales of Infant and Toddler Development 3rd edition

Adjustment variables were sex, age in months, whether the child lives with a young sibling (< 5 years), maternal characteristics (age group, health insurance, educational status, occupational status, marital status and parity), and household characteristics (wealth index category, Indigenous status, affiliation to social programs and reported category of monthly income from all sources). Standard errors were adjusted for data dependencies within communities

^a^ Wasting (weight for height Z < -2) was not analyzed since there were few events and there was no outcome variation within some covariate categories

^b^ Height for age Z < -2

^c^ Weight for age Z < -2

^d^ Weight for height Z > 2

Sample size for nutritional status and EDI-II outcomes: 1060

Sample size for BSID-III outcomes: 554

### Associations between maternal depressive symptomatology and child neurodevelopment

Children whose mothers had clinically significant depressive symptomatology had lower odds of normal neurodevelopment as assessed by the EDI-II test (OR = 0.79 95%CI: 0.63, 0.99), lower BSID-III cognitive composite scores (MD=-1.73 95%CI: − 3.26, − 0.20; Cohen’s d= − 0.16 95%CI: − 0.30, − 0.02) and lower BSID-III socioemotional composite scores (MD = − 3.15 95%CI: − 5.59, − 0.70; Cohen’s d = − 0.25 95%CI: − 0.44, − 0.05). After covariate adjustment, the categorical maternal depressive symptomatology exposure remained associated with the socioemotional composite scores (AMD=-2.31 95%CI: − 4.49, − 0.13), the ATT was similar in magnitude and also statistically significant (Table 3). There were no statistically significant associations with the indicators of impaired neurodevelopment nor with the subtest scaled scores.

### Sex-specific associations between maternal depressive symptomatology and nutritional status or child development outcomes

In male children, those whose mothers had clinically significant depressive symptomatology had 40% lower odds of normal neurodevelopment (AOR = 0.60 95%CI:0.41, 0.88) compared to other children, and this association was statistically different between sexes (*p* = 0.044, Table 4). In female children whose mothers had clinically significant depressive symptomatology, the covariate-adjusted mean of the socioemotional composite score (BSID-III) was lower compared to other female children (AMD = − 3.13 95%CI: − 6.11, − 0.15). There were no sex-specific statistically significant associations for the other outcomes (Table 4). Associations with the standardized CES-D scores are presented in Supplementary Material Table ST5. Female children of mothers with clinically significant depressive symptomatology had 7.6% points (95%CI: 0.1, 15.0) higher prevalence of stunting compared to a situation in which this maternal depressive symptomatology condition is absent (ATT in Supplementary Material Table ST6).

**Table 4:** Covariate-adjusted associations between maternal clinically significant depressive symptomatology and child development outcomes by sex and household Indigenous status among children aged 0 to 38 months from 24 communities in Oaxaca Mexico

	Sex			Household Indigenous status			
	Males	Females		Non-Indigenous	Indigenous without Indigenous language spoken	Indigenous with Indigenous language spoken	
	AOR (95%CI)		*P*	AOR (95%CI)			*P*
*Nutritional status* ^*a*^							
Stunting^b^	1.21 (0.87, 1.69)	1.47 (0.92, 2.34)	0.554	1.50* (1.05, 2.16)	1.64* (1.04, 2.60)	0.81 (0.58, 1.15)	0.003
Underweight^c^	1.18 (0.62, 2.24)	1.42 (0.71, 2.85)	0.722	2.10* (1.10, 4.01)	0.90 (0.35, 2.30)	0.62 (0.19, 2.02)	0.086
*Child Development Evaluation Test 2nd Edition – EDI-II*							
Normal neurodevelopment	0.60** (0.41, 0.88)	1.22 (0.73, 2.05)	0.044	1.10 (0.68, 1.78)	0.70 (0.36, 1.36)	0.81 (0.59, 1.13)	0.523
	AMD (95% CI)		*P*	AMD (95% CI)			*P*
*Bayley Scales of Infant and Toddler Development 3rd edition (BSID-III) composite scores*							
Cognitive	-0.57 (-3.17, 2.02)	-0.05 (-2.52, 2.41)	0.759	0.60 (-2.64, 3.84)	-0.20 (-2.84, 2.43)	-1.31 (-3.92, 1.30)	0.639
Language	-1.38 (-4.97, 2.21)	-1.47 (-4.84, 1.91)	0.968	-1.22 (-6.39, 3.96)	-0.15 (-3.47, 3.17)	-3.33** (-5.74, -0.93)	0.335
Motor	-0.44 (-3.74, 2.86)	1.87 (-2.13, 5.86)	0.370	0.62 (-4.98, 6.22)	2.03 (-1.81, 5.88)	-0.64 (-3.08, 1.80)	0.527
Socio-emotional	-1.36 (-4.44, 1.73)	-3.13* (-6.11, -0.15)	0.379	-2.17 (-7.05, 2.71)	-3.04* (-5.92, -0.16)	-1.49 (-4.15, 1.17)	0.758
*Bayley Scales of Infant and Toddler Development 3rd edition (BSID-III) subtest scaled scores*							
Receptive language	-0.30 (-0.98, 0.38)	-0.23 (-0.90, 0.44)	0.899	-0.12 (-1.02, 0.78)	-0.15 (-0.84, 0.55)	-0.56* (-1.09, -0.02)	0.565
Expressive language	-0.15 (-0.75, 0.44)	-0.29 (-0.86, 0.29)	0.662	-0.29 (-1.29, 0.72)	0.09 (-0.42, 0.60)	-0.58** (-0.97, -0.20)	0.131
Fine motor	-0.23 (-0.91, 0.45)	0.35 (-0.39, 1.08)	0.239	0.09 (-1.18, 1.36)	0.18 (-0.51, 0.86)	-0.05 (-0.73, 0.62)	0.877
Gross motor	0.09 (-0.55, 0.73)	0.27 (-0.42, 0.95)	0.695	0.14 (-0.66, 0.94)	0.48 (-0.17, 1.14)	-0.17 (-0.79, 0.46)	0.363
*Impaired neurodevelopment (BSID-III composite scores < 85)*							
	AOR (95%CI)		P	AOR (95%CI)			P
Cognitive	1.97 (0.76, 5.11)	0.82 (0.41, 1.66)	0.109	1.51 (0.48, 4.74)	0.79 (0.26, 2.37)	1.63 (0.78, 3.41)	0.556
Language	1.04 (0.58, 1.87)	1.05 (0.46, 2.38)	0.982	1.41 (0.57, 3.46)	0.79 (0.40, 1.53)	1.08 (0.66, 1.78)	0.433
Motor	1.66 (0.87, 3.17)	0.59 (0.25, 1.37)	0.074	1.39 (0.47, 4.11)	0.64 (0.27, 1.56)	1.18 (0.63, 2.19)	0.355

*p < 0.05, **p < 0.01, ***p < 0.001

Maternal clinically significant depressive symptomatology was defined as a Center for Epidemiologic Studies Depression scale (CES-D) score of 16 or higher,

CI: Confidence interval

AOR: covariate adjusted odds ratio (CES-D ≥ 16 vs. CES-D < 16). AMD: covariate adjusted mean difference (CES-D ≥ 16 vs. CES-D < 16)

Adjustment variables were sex, age in months, whether the child lives with a young sibling (< 5 years), mother characteristics (age group, health insurance, educational status, occupational status, marital status and parity), and household characteristics (wealth index category, Indigenous status, affiliation to social programs and reported category of monthly income from all sources). Standard errors were adjusted for data dependencies within communities

^a^Wasting (weight for height Z < -2) and overweight or obesity (weight for height Z > 2) indicators were not analyzed since the number of outcome events was zero or very low within subgroup-exposure categories

^b^Height for age Z < -2

^c^Weight for age Z < -2

Sample size for nutritional status and EDI-II outcomes: 1060 (567 males and 493 females; 360 from non-Indigenous households, 436 from Indigenous households without Indigenous language spoken, 264 from Indigenous households with Indigenous language spoken)

Sample size for BSID-III outcomes: 554 (269 males and 285 females; 166 from non-Indigenous households, 241 from Indigenous households without Indigenous language spoken, 147 from Indigenous households with Indigenous language spoken)

### Household Indigenous status specific associations between maternal depressive symptomatology and nutritional status or child development outcomes

Maternal clinically significant depressive symptomatology was associated with higher covariate-adjusted odds of stunting among children from non-Indigenous households and children from Indigenous households in which none of the parents spoke an Indigenous language, in contrast, this association was not significant for children living in households with an Indigenous language speaking parent. The joint test of significance revealed heterogeneity across these associations (*p* = 0.003, Table 4). In non-Indigenous households, the odds of underweight doubled in children whose mothers had clinically significant depressive symptoms compared to other children (AOR = 2.10 95%CI: 1.10, 4.01). Language BSID-III scores were lower with maternal clinically significant depressive symptomatology among children from Indigenous language speaking households (Table 4). 

### Associations between other covariates and child development outcomes

Regarding social determinants of health, we found that better child development outcomes were observed among children whose mothers had higher education or were married (as compared to single), lived in households with a higher wealth index or higher income, in some of our analyses (Supplementary tables ST7-ST9).

### Complete case analysis

Results based on the complete case analysis and using the transformed CES-D variable are shown in Supplementary Material (Tables ST10-ST12). In general, the same pattern of results was observed.

## Discussion

We found that maternal depressive symptomatology was associated with higher odds of stunting, higher odds of underweight, lower means of cognitive and socioemotional BISID-III composite scores among children aged 0 to 38 months from 24 communities in Oaxaca Mexico. These results are concordant with previous international research. For example, a meta-analysis performed by Liu and cols. (2017) found lower cognitive development scores when comparing children of mother with high vs. low depressive symptoms, the magnitude of the mean difference was higher to the one we found (Cohen’s d = − 0.25 vs. − 0.16), however, the aforementioned study was based on data mostly from high income countries. Along the same lines, Fan and cols. (2013) found an association of similar magnitude to ours (Cohen’s d = − 0.19, 95%CI: − 0.30, − 0.08) among 0–2-year-olds using studies from middle- and high-income countries. International evidence on the socioemotional domain was more limited, Fan and cols. (2013) estimated an association based on 3 studies from high income countries that used the BSID-III instrument (Cohen’s d = − 0.18, 95%CI: − 0.29, -0.06). Our association between maternal depressive symptomatology status and the socioemotional composite scores expressed as Cohen’s d was − 0.25 (95%CI: − 0.44, − 0.05).

All the previous results and comparisons refer to crude associations. Once we adjusted for multiple general and sociodemographic characteristics, the associations between maternal depressive symptomatology and stunting remained statistically significant. A meta-analysis from 11 low and middle income countries also found a significant association with stunting (OR = 1.4; 95% CI: 1.2, 1.7) [52] which was similar in magnitude to our study (OR = 1.32; 95%CI: 1.04, 1.69). In contrast, nationally representative data from Mexico in 2012 showed no significant differences in the prevalence of nutritional status indicators between children of mothers with and without depressive symptomatology, as measured by a validated reduced version of the CES-D scale (15.8% vs. 14.7% for stunting, 4.1% vs. 3.2% for underweight, and 10.5% vs. 9.7% for overweight) [13, 50]. Differences with respect to these results may stem from the heterogeneity in subpopulations within Mexico. Our sample was obtained from Oaxaca, one of the three states of the country with the lowest human development index and with the highest percentage of Indigenous population (Instituto Nacional de Estadística y Geografía, [30]; Programa de las Naciones Unidas Para el Desarrollo, [42])

Except for the socioemotional composite scores, associations between maternal depressive symptomatology and child development outcomes from the EDI-II and BSID-III tests were not statistically significant in the overall sample after covariate adjustment. However, there were statistically significant associations when we estimated sex specific and household Indigenous status specific associations.

Male children whose mothers had clinically significant depressive symptomatology had 40% lower odds of normal neurodevelopment based on the EDI-II test (AOR = 0.60, 95%CI:0.41, 0.88), in contrast, no such difference was found in female children. There are few studies analyzing whether associations between maternal depression and child development outcomes vary by sex. Our results align with findings from a recent meta-analysis based on 12 studies from six countries (UK, Canada, Australia, South Africa, USA, and Finland) in which maternal depression was significantly associated with child and adolescent cognitive outcomes in males but not in females [1]. It is possible that the maturational advantage experienced in females may act as a protective factor against postpartum depression, or that depressed mothers treat their male and female children differently [23]. More research is needed to better understand the mechanisms behind sex differences in the associations of maternal depression with child development outcomes.

The analysis of covariate-adjusted associations by household Indigenous status revealed that our two exposures of maternal depressive symptomatology were associated to lower language scores among children who lived in Indigenous households with and Indigenous language speaking parent. There is a dearth of evidence on these associations for Indigenous populations. One study performed in rural Mexico found that maternal depressive symptoms was associated with more child behavior problems, this association was stronger for children living in Indigenous households [19].

Multiple mechanisms that could explain a causal link between maternal depression and child development have been identified. Two of them are feeding practices and early child stimulation [16, 22, 29, 32]. The presence of maternal depressive symptomatology can result in lowered functional capacity, reducing the quality of childcare practices and therefore increasing the risk of child development deficits. Failing to provide necessary nutrients can negatively impact children’s physiological and neurological development. It is important to note that child growth and cognition are specially sensitive to nutrient effects during the first 1000 days of life [6]. Another mechanism that can compromise children’s neurodevelopment is a lowered quality of mother-child interactions, maternal depression has been associated with insensitive engagement with the child [11]. There is also evidence documenting a strong association between perinatal mental health problems and child maltreatment [3]. The future mental health of the child can also be affected, epigenetic studies in humans have shown a link between emotional neglect and depression later in life [34].

It is also possible that postpartum maternal depression and compromised child development are both reflecting exposure to maternal depression prenatally. Fetal adaptation to adverse conditions may lead to changes in physiologic functions, leaving the brain or body vulnerable with long-lasting consequences, this concept is referred to as fetal programming [15, 28]. There is a proposed developmental model on the mechanisms of transmission from maternal depression to children’s psychopathology that includes heritability of depression and exposure to stressful environment [21].

### Recommendations

Public health interventions can be set to target mothers at risk of developing depression through screening. Preventing maternal depression is very important since the responsive and affective involvement of mothers in childcare will enable them to better detect their children’s emotional and physiological needs and provide better care practices. Maternity blues has been identified as an important risk factor for developing maternal postpartum depression; the first weeks after delivery are a key period to detect the appearance of this condition and provide early care [46]. Detecting maternal depression requires proper treatment management that may include psychotherapy in general and prescribed medication when depression is severe [34]. However, there are concerns about prescribing medication to women who are breastfeeding, given the possible adverse effects on infants through breast milk [41]. Adequate treatment of depression requires health services with properly trained physicians and health workers at the primary healthcare level to monitor and evaluate maternal health. Health systems in low- and middle-income countries must prepare to effectively address this potentially growing health problem, while considering the sociocultural contexts in different regions. It is imperative to develop tools in this field to detect cases in a timely manner and strengthen primary care units. An integrated approach has been proposed to addresses both maternal depression and parenting behaviors that promote healthy development among children [20].

### Strengths and limitations

The present study has strengths, including a moderate to large sample size, child development and anthropometry measurements gathered by well-trained personnel, and the ability to adjust for several sociodemographic characteristics of mothers and their households. We were able to increase the analysis sample size from 886 to 1060 mother-child dyads (99% of the initial sample in which the mother was the primary caregiver) using the MICE methodology which requires the missingness at random (MAR) assumption, this means that missingness does not depend on unobserved variables after conditioning on the observed variables. There is no way to determine whether the MAR assumption is true based solely on the data, however, this assumption becomes more plausible by adding more explanatory variables in the analysis [54].

The main limitation of the study is its cross-sectional and observational nature. Therefore, it is not possible to determine causality from the data. The timeframe of the instruments and the time it takes for a cause to produce an effect further complicates the assessment of the studied relationships. The CES-D instrument asks about the frequency of depressive symptoms during the past week, whereas the child neurodevelopment and nutritional status outcomes that we analyzed may vary in terms of the time frame they capture. For example, stunting is an indicator of chronic undernutrition and external shocks occurring early in life can have long-lasting consequences. There is also potential bidirectionality in the relationship between maternal depressive symptomatology and our outcome variables, measuring both variables at multiple time points in the same mother-child dyads could help determining directionality. Another limitation is the potential bias in reporting. Child development indicators that are not measured through direct observation of the child, but rather, are based on information provided by the mother, are susceptible to bias that may occur along with bias in reporting of depressive symptoms. Most of our indicators were based on direct observations of children’s behavior or anthropometric measurements. One exception was the socioemotional BSID-III score. Regarding the estimation of group specific associations, our statistical power was low to detect associations of small magnitude, especially for the analysis in the subsample with BSID-III scores. An additional limitation was the lack of a measurement of prenatal depressive symptoms and whether pregnancy was unplanned. Finally, although we included multiple sociodemographic maternal and household characteristics as adjustment variables, the possibility that omitted variables act as confounders cannot be completely ruled out.

We used data from a study originally planned as a stepped wedge design to evaluate an educational intervention on early child development [43]. It was stopped in March 2020 due to the COVID-19 emergency, but baseline data was obtained by then from 24 communities. The emergency highlighted the importance and need to target the most vulnerable who lack mechanisms to cope with external shocks that can worsen maternal mental health and hamper children’s development [18].

## Conclusion

Maternal depressive symptomatology was associated with stunting, lower odds of normal neurodevelopment in male children and worsen language outcomes in children from Indigenous households in Oaxaca Mexico.

## Supplementary Information

Below is the link to the electronic supplementary material.

Supplementary Material 1

Supplementary Material 2

## Data Availability

The research data supporting the results of this manuscript is available as Online Resource material Stata v.19 file (SM2_data.dta).
