# Supplementary material for: Maternal depressive symptomatology and its association with early child development in socioeconomically deprived Mexican households
Source: Discov Ment Health. 2026 May 19;6(1):133. doi: 10.1007/s44192-026-00477-6 (PMC13357464; doi:10.1007/s44192-026-00477-6)
Supplement: Supplementary file 1 — Supplementary Material 1 [file 44192_2026_477_MOESM1_ESM.pdf]

SUPPLEMENTARY MATERIAL

Title: Maternal depressive symptomatology and its association with early child development in socioeconomically deprived Mexican households

Amado D. Quezada-Sánchez[1,2]*, Miia S. Vesterinen[1], Tarja I. Kinnunen[1], Carmen Hernández-Chávez[3], Angélica García-Martínez[4], Abby Madrigal-Ramírez[5], Raquel García-Feregrino[2], Evelyn Fuentes-Rivera[6], Armando García-Guerra[7], Edson Serván-Mori[5].

Journal: Discover Mental Health

Affiliations

1. Unit of Health Sciences, Faculty of Social Sciences. Tampere University, Tampere, Finland
2. Center for Evaluation and Survey Research, National Institute of Public Health, Cuernavaca, Morelos, México
3. Department of Developmental Neurobiology, National Institute of Perinatology Isidro Espinosa de los Reyes, Mexico City, México
4. Lucy Family Institute for Data and Society, University of Notre Dame, Notre Dame, IN, USA
5. Center for Health Systems Research, National Institute of Public Health, Cuernavaca, Morelos, México.
6. Center for Demographic, Urban and Environmental Studies, College of Mexico A.C., Mexico City, México.
7. Center for Nutrition and Health Research. National Institute of Public Health, Cuernavaca, Morelos, México.

*Corresponding author e-mail: amado.quezadasanchez@tuni.fi

CONTENTS

Table ST1. Household wealth index construction by Principal Component analysis.......................... 3

Missing data analysis and Multiple imputation by chained equations (MICE) 4

Table ST2. Missing data patterns and sample sizes 5

Table ST3. Sociodemographic characteristics and maternal depressive symptomatology between dyads included and not included in the complete case analysis.. 6

Figure SF1. Distribution of maternal CES-D scores in the analysis sample.. 8

Table ST4. Distribution of general and sociodemographic characteristics of study sample by household Indigenous status 9

Table ST5. Covariate-adjusted associations between maternal standardized CES-D scores and child development outcomes by sex and household Indigenous status among children aged 0 to 38 months from 24 communities in Oaxaca Mexico 11

Table ST6. Average treatment effects on the treated for comparing children whose mothers had clinically significant depressive symptomatology with their estimated counterfactual of no maternal clinically significant depressive symptomatology 13

Table ST7. Multiple logistic regressions for child nutritional status and the EDI-II neurodevelopment normal result among 1060 children aged 0 to 38 months from 24 communities in Oaxaca Mexico.. 14

Table ST8. Multiple linear regression models for Bayley Scales of Infant and Toddler Development 3rd edition composite scores in a subsample of 554 children from 24 communities in Oaxaca Mexico 16

Table ST9. Multiple linear regression models for Bayley Scales of Infant and Toddler Development 3rd edition subtest scaled scores in a subsample of 554 children from 23 communities in Oaxaca Mexico 18

Table ST10. Covariate-adjusted associations between clinically significant depressive symptomatology and child development outcomes from complete case analyses 20

Table ST11. Covariate-adjusted associations between CES-D standardized scores and child development outcomes from complete case analyses... 21

Table ST12. Covariate-adjusted associations between the transformed scale of maternal depressive symptomatology and nutritional status and neurodevelopment outcomes among children aged 0 to 38 months from 24 communities in Oaxaca Mexico 22

Table ST1. Household wealth index construction by Principal Component analysis

Variables	Correlation with First Principal Component	Score weight[a]
**House materials**		
Floor[b]	0.45	0.15
Walls[c]	0.58	0.19
Roof[d]	0.58	0.19
**House services**		
Gas stove	0.58	0.19
Water supply[e]	0.42	0.14
Toilet[f]	0.55	0.19
**Possession of goods**		
Automobile	0.34	0.12
Television or computer	0.49	0.16
Refrigerator, washing machine, dryer, stove, and furniture	0.62	0.21
Electric appliances	0.58	0.20
Mobile or fixed Phone line	0.47	0.16

n=1060. The first principal component absorbed 27.0% of the total variance.
[a]Score weight for standardized variables
[b]Coded as 0=Dirt; 1=Concrete; 2= Floor covering e.g. mosaic.
[c]Coded as 0=provisional materials, cardboard sheet, asbestos or metal sheet, reed/bamboo/palm, wood, adobe; 1=Concrete, block, quarry, stone
[d]Coded as 0=provisional materials, carboard/asbestos/metal sheet, wood, roof with beams, tile; 1= Concrete.
[e]Coded as 0=other source e.g. well, river, or a lake; 1=running water outside the house but in the property; 2 = running water inside the house.
[f]Coded as 0=other; 1=toilet connected to drainage or a septic tank.
Barlett test of sphericity: chi2(55df) =1991.9, p<0.001. Kaiser-Meyer-Olkin measure of sampling adequacy = 0.774

Missing data analysis and Multiple imputation by chained equations (MICE)

The initial sample size consisted of 1073 mother-child dyads in which the mother was the primary caregiver. We excluded 2 observations with missing or inconsistent maternal age and 1 observation with out-of-range child age. We then identified a total of 10 variables in which there were <0.5% of missing values. These variables and their number of missing values in parentheses were sex (0), child's age group (0), whether there was a <5-year-old sibling living in the household (0), maternal age group (0), maternal completed education level (2), type of health insurance (3), occupational status (2), marital status (3), household Indigenous status (0), and number of social programs the household was affiliated with (0). We excluded dyads with missing data in any of these variables resulting in a total sample size of 1060 dyads.

The analysis of missing data patterns for the analysis variables in this sample of n=1060 dyads is shown in Table ST2. A total of 867 observations had complete information in all variables, 886 dyads were included in complete case analysis because they had complete information on the exposure variable and covariates, and one of the outcomes (nutritional status or EDI-II result). The variable with the highest number of missing values was household income (96 missing values, 9.1%) and the incomplete variable with the least missing values (24, 2.3%) was the wealth index.

Table ST2. Missing data patterns and sample sizes

	Sample size	Outcomes		Exposure	Covariates		
		Nutritional Status	EDI-II result	CES-D	Income	Parity	Wealth Index
Included in complete case analysis	867	Present	Present	Present	Present	Present	Present
	11	Present	Missing	Present	Present	Present	Present
	8	Missing	Present	Present	Present	Present	Present
Not included in complete case analysis	58	Present	Present	Present	Missing	Present	Present
	30	Present	Present	Missing	Present	Present	Present
	5	Present	Present	Missing	Missing	Present	Present
	26	Present	Present	Present	Present	Missing	Present
	4	Present	Present	Present	Missing	Missing	Present
	1	Present	Present	Missing	Missing	Missing	Present
	14	Missing	Missing	Present	Present	Present	Present
	4	Missing	Missing	Present	Missing	Present	Present
	2	Missing	Missing	Missing	Present	Present	Present
	1	Missing	Missing	Present	Present	Missing	Present
	5	Missing	Missing	Missing	Missing	Missing	Present
	3	Present	Present	Present	Present	Present	Missing
	6	Present	Present	Present	Missing	Present	Missing
	2	Present	Present	Missing	Present	Present	Missing
	5	Present	Present	Missing	Missing	Present	Missing
	1	Present	Present	Present	Missing	Missing	Missing
	3	Present	Present	Missing	Missing	Missing	Missing
	1	Missing	Present	Present	Missing	Present	Missing
	1	Missing	Present	Missing	Missing	Missing	Missing
	1	Missing	Missing	Missing	Missing	Present	Missing
	1	Missing	Missing	Missing	Missing	Missing	Missing
Total	1060						
Number of Missing values for each variable		38	39	56	96	43	24

Using the 10 variables with complete information as predictors we performed multiple imputations by chained equations (MICE) with m=20 repetitions. We used logistic regression for binary variables, truncated linear regression for the CES-D, linear regression for nutritional status Z scores, Poisson regression for parity centered at 1 (its minimum value), and ordinal regression for the EDI-II result, category of wealth index and category of income. We repeated

the procedure for the analyses using clinically significant depressive symptomatology as exposure variable (instead of the CES-D score).

Dyads not included in the complete case analysis had younger child ages and were more likely to have a sibling <5 years old living in the same household. They also had higher mean of maternal CES-D scores, a higher percentage of mothers with a job, a higher mean parity and a higher percentage living in non-Indigenous households, compared to those included in the complete case analysis (Table ST3).

Table ST3. Sociodemographic characteristics and maternal depressive symptomatology between dyads included and not included in the complete case analysis

		Complete case analysis		
	Total	Not Included	Included	P
**Child characteristics**				
Sex				
Male	567 (53.5%)	96 (55.2%)	471 (53.2%)	0.464
Female	493 (46.5%)	78 (44.8%)	415 (46.8%)	
Child's age in months	19.6 (9.0)	19.6 (10.6)	19.6 (8.7)	0.938
Child's age group in months				
0 to 11	253 (23.9%)	49 (28.2%)	204 (23.0%)	0.021
12 to 23	422 (39.8%)	54 (31.0%)	368 (41.5%)	
24 to 38	385 (36.3%)	71 (40.8%)	314 (35.4%)	
Has a sibling <5 years of age				
No	733 (69.2%)	105 (60.3%)	628 (70.9%)	0.022
Yes	327 (30.8%)	69 (39.7%)	258 (29.1%)	
**Maternal characteristics**				
Maternal depressive symptomatology CES-D				
CES-D score	12.7 (9.9)	16.1 (12.1)	12.2 (9.5)	0.023
CES-D<16	727 (72.4%)	68 (57.6%)	659 (74.4%)	0.006
CES-D≥16	277 (27.6%)	50 (42.4%)	227 (25.6%)	
Missing, n	56			
Age group in years				
15 to 19	95 (9.0%)	23 (13.2%)	72 (8.1%)	0.138
20 to 29	606 (57.2%)	95 (54.6%)	511 (57.7%)	
30 to 49	359 (33.9%)	56 (32.2%)	303 (34.2%)	
Completed education level				
Elementary or none	234 (22.1%)	38 (21.8%)	196 (22.1%)	0.683
Middle	459 (43.3%)	82 (47.1%)	377 (42.6%)	
High school	260 (24.5%)	37 (21.3%)	223 (25.2%)	

College/Univeristy or Professional	107 (10.1%)	17 (9.8%)	90 (10.2%)	
Health insurance				
None	101 (9.5%)	18 (10.3%)	83 (9.4%)	
Seguro Popular	839 (79.2%)	133 (76.4%)	706 (79.7%)	0.560
Social security	120 (11.3%)	23 (13.2%)	97 (10.9%)	
Occupation status				
Has a job	215 (20.3%)	51 (29.3%)	164 (18.5%)	
Housework	450 (42.5%)	65 (37.4%)	385 (43.5%)	
Housework with extra activities	158 (14.9%)	22 (12.6%)	136 (15.3%)	0.031
Self-employed	204 (19.2%)	29 (16.7%)	175 (19.8%)	
Other	33 (3.1%)	7 (4.0%)	26 (2.9%)	
Marital status				
Single	95 (9.0%)	16 (9.2%)	79 (8.9%)	
Free Union	567 (53.5%)	93 (53.4%)	474 (53.5%)	0.468
Married	347 (32.7%)	52 (29.9%)	295 (33.3%)	
Separated/Divorced/Widowed	51 (4.8%)	13 (7.5%)	38 (4.3%)	
Parity	2.07 (1.14)	2.31 (1.54)	2.03 (1.06)	0.045
Missing, n	43			
**Household characteristics**				
3 quantiles of sesn				
Low	346 (33.4%)	63 (42.0%)	283 (31.9%)	
Medium	346 (33.4%)	43 (28.7%)	303 (34.2%)	0.069
High	344 (33.2%)	44 (29.3%)	300 (33.9%)	
Missing, n	24			
Reported monthly income				
Less than 189€	416 (43.2%)	38 (48.7%)	378 (42.7%)	
189€ to 314€	345 (35.8%)	25 (32.1%)	320 (36.1%)	0.568
>314€ to 440€	108 (11.2%)	6 (7.7%)	102 (11.5%)	
More than 440€	95 (9.9%)	9 (11.5%)	86 (9.7%)	
Missing, n	96			
Indigenous status				
Non-Indigenous	360 (34.0%)	83 (47.7%)	277 (31.3%)	
Indigenous without Indigenous language spoken	436 (41.1%)	44 (25.3%)	392 (44.2%)	<0.001
Indigenous with Indigenous language spoken	264 (24.9%)	47 (27.0%)	217 (24.5%)	
Number of social programs				
None	759 (71.6%)	120 (69.0%)	639 (72.1%)	
One	241 (22.7%)	40 (23.0%)	201 (22.7%)	0.541
Two to four	60 (5.7%)	14 (8.0%)	46 (5.2%)	

Frequency (percentage) or mean (SD) are presented

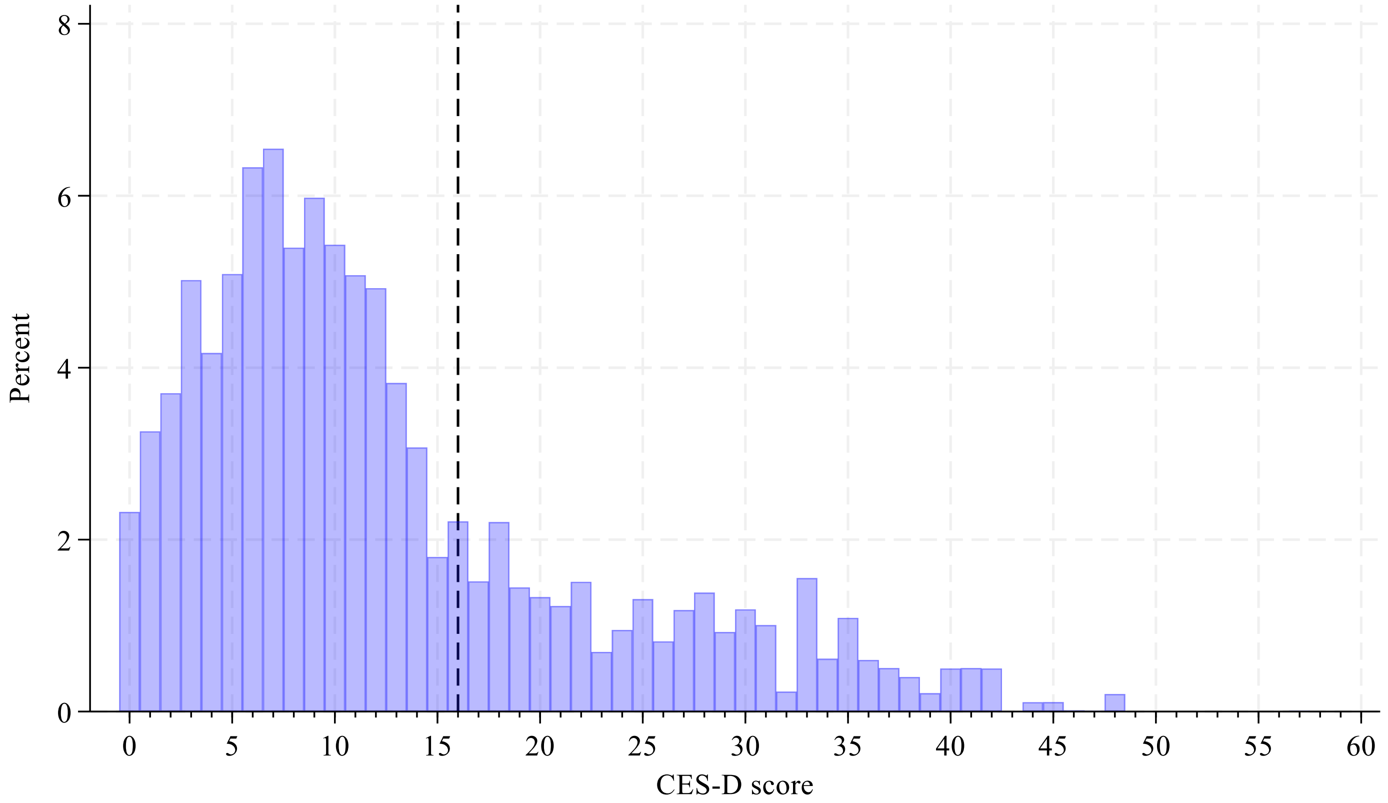

Figure SF1. Distribution of maternal CES-D scores in the analysis sample

CES-D: Center for Epidemiologic Studies depression scale.
The reference line at CES-D=16 corresponds to the threshold defining the two-level categorical exposure (CES-D <16, CES-D ≥16)
n=1060, mean = 12.7, Median = 10, Standard Deviation = 10.0, non-parametric skewness = 0.27
56 missing values were imputed m=20 times using multiple imputations with chained equations.

Table ST4. Distribution of general and sociodemographic characteristics of study sample by household Indigenous status

	Type of household			
	Non-Indigenous (n=360)	Indigenous without Indigenous language spoken (n=436)	Indigenous with Indigenous language spoken (n=264)	Total (n=1060)
**Child characteristics**				
Female, %	45	49.1	44.3	46.5
Age in months, mean (SD)	19.0 (8.8)	20.0 (9.3)	20.0 (8.8)	19.6 (9.0)
Age group in months, %				
0 to 11	27.2	22.9	20.8	23.9
12 to 23	38.3	38.8	43.6	39.8
24 to 38	34.4	38.3	35.6	36.3
Has a sibling <5 years of age, %	33.3	29.4	29.9	30.8
**Maternal characteristics**				
Age group in years, %				
15 to 19	12.5	8.3	5.3	9.0
20 to 29	58.9	58.3	53	57.2
30 to 39	28.6	33.5	41.7	33.9
Completed education level, %				
None or elementary	14.4	17	40.9	22.1
Middle school	44.2	48.9	33.0	43.3
Highschool	25.6	26.6	19.7	24.5
College/University	15.8	7.6	6.4	10.1
Health insurance, %				
None	11.4	9.4	7.2	9.5
Seguro Popular	75	78.7	85.6	79.2
Social Security	13.6	11.9	7.2	11.3
Occupation status, %				
Has a job	16.7	22.9	20.8	20.3
Housework	40.6	38.5	51.5	42.5
Housework with extra activities[a]	13.6	18.3	11.0	14.9
Self-employed	24.7	17.7	14.4	19.2
Other[b]	4.4	2.5	2.3	3.1
Marital status, %				
Single	11.4	9.4	4.9	9.0
Free Union	51.9	53.4	55.7	53.5
Married	31.4	32.6	34.8	32.7

Separated/Divorced/Widowed	5.3	4.6	4.5	4.8
Parity, number of times has given birth to a live neonate[c], mean (SD)	1.98 (1.00)	2.08 (1.25)	2.14 (1.11)	2.06 (1.14)
**Household characteristics**				
Wealth index category[d], %				
Low	30.1	32.6	38.9	33.3
Medium	30.8	36.4	32.2	33.4
High	39.2	31.0	28.9	33.2
Reported monthly income[e]				
Less than 189€	34.6	44.3	53.7	43.3
189€ to 314€	38.6	36.5	30.6	35.7
>314€ to 440€	13.9	10.9	7.9	11.2
More than 440€	13.0	8.3	7.8	9.8
Number of social programs, %				
None	73.6	72.5	67.4	71.6
One	20.8	21.6	27.3	22.7
Two to four	5.6	6.0	5.3	5.7

[a] Extra activities such as helping in a business or selling a product.
[b] Includes being a student or retired.
[c] 43 missing values were imputed using MICE in each repetition.
[d] 24 missing values were imputed using MICE in each repetition.
[e] Converted from Mexican pesos to euros using the 2020 purchase power parity exchange rates for private consumption, 96 missing values were imputed using MICE in each repetition.

Table ST5. Covariate-adjusted associations between maternal standardized CES-D scores and child development outcomes by sex and household Indigenous status among children aged 0 to 38 months from 24 communities in Oaxaca Mexico

	Sex			Household Indigenous status			
	Males	Females		Non-Indigenous	Indigenous without Indigenous language spoken	Indigenous with Indigenous language spoken	
	AOR (95%CI)		P		AOR (95%CI)		P
**Nutritional status**[a]							
Stunting[b]	1.03 (0.88, 1.20)	1.16 (0.92, 1.46)	0.448	1.11 (0.93, 1.31)	1.28* (1.02, 1.59)	0.85 (0.69, 1.04)	0.005
Underweight[c]	1.12 (0.86, 1.45)	1.06 (0.81, 1.39)	0.775	1.17 (0.91, 1.52)	1.07 (0.76, 1.51)	0.92 (0.56, 1.51)	0.686
**Child Development Evaluation Test 2nd Edition – EDI-II**							
Normal neurodevelopment	0.84 (0.68, 1.05)	1.05 (0.81, 1.37)	0.290	1.08 (0.89, 1.32)	0.86 (0.65, 1.14)	0.84* (0.71, 0.98)	0.101
	AMD (95% CI)		P		AMD (95% CI)		P
**Bayley Scales of Infant and Toddler Development 3rd edition (BSID-III) composite scores**							
Cognitive	-0.11 (-1.45, 1.23)	-0.10 (-1.25, 1.05)	0.990	0.32 (-0.96, 1.61)	-0.15 (-1.21, 0.92)	-0.55 (-1.90, 0.80)	0.631
Language	-0.57 (-2.07, 0.94)	-0.23 (-1.88, 1.41)	0.730	0.11 (-1.85, 2.07)	-0.10 (-1.85, 1.64)	-1.39* (-2.56, -0.22)	0.382
Motor	0.63 (-0.97, 2.23)	0.54 (-0.97, 2.05)	0.936	0.97 (-0.91, 2.86)	0.81 (-0.93, 2.56)	-0.21 (-1.58, 1.16)	0.398
Socio-emotional	-1.04 (-2.62, 0.55)	-1.34 (-3.18, 0.50)	0.798	-0.18 (-2.29, 1.92)	-1.54 (-3.33, 0.25)	-1.92* (-3.75, -0.09)	0.322
**Bayley Scales of Infant and Toddler Development 3rd edition (BSID-III) subtest scaled scores**							
Receptive language	-0.12 (-0.42, 0.17)	-0.03 (-0.39, 0.33)	0.666	0.04 (-0.32, 0.41)	-0.08 (-0.45, 0.30)	-0.21 (-0.51, 0.10)	0.557

Expressive language	-0.06 (-0.31, 0.20)	-0.06 (-0.31, 0.20)	0.991	0.01 (-0.35, 0.37)	0.04 (-0.22, 0.30)	-0.27** (-0.43, -0.11)	0.137
Fine motor	-0.03 (-0.35, 0.28)	0.13 (-0.20, 0.45)	0.511	0.07 (-0.28, 0.41)	0.09 (-0.31, 0.49)	-0.01 (-0.37, 0.35)	0.900
Gross motor	0.24 (-0.05, 0.54)	0.05 (-0.20, 0.29)	0.322	0.26 (-0.08, 0.60)	0.18 (-0.03, 0.38)	-0.06 (-0.26, 0.14)	0.087
**Impaired neurodevelopment (BISID-III composite scores <85)**							
	AOR (95%CI)		P		AOR (95%CI)		P
Cognitive	1.20 (0.80, 1.80)	0.94 (0.66, 1.33)	0.314	1.08 (0.67, 1.73)	0.74 (0.45, 1.21)	1.36 (0.97, 1.91)	0.233
Language	0.98 (0.70, 1.36)	1.01 (0.70, 1.47)	0.903	1.04 (0.75, 1.46)	0.93 (0.60, 1.44)	1.02 (0.79, 1.30)	0.892
Motor	1.00 (0.69, 1.46)	0.82 (0.59, 1.13)	0.355	0.95 (0.60, 1.51)	0.78 (0.48, 1.28)	0.99 (0.69, 1.42)	0.738

*p<0.05, **p<0.01, ***p<0.001

CI: Confidence interval. CES-D: Center for Epidemiologic Studies Depression Scale. The CES-D was standardized with the overall sampling mean (12.7) and standard deviation (10.0).

AOR: covariate adjusted odds ratio (CES-D+1SD vs CES-D). AMD: covariate adjusted mean difference (CES-D+1SD vs CES-D).

Adjustment variables were sex, age in months, whether the child lives with a young sibling (<5 years), mother characteristics (age group, health insurance, educational status, occupational status, marital status and parity), and household characteristics (wealth index category, Indigenous status, affiliation to social programs and reported category of monthly income from all sources). Standard errors were adjusted for data dependencies within communities.

[a] Wasting (weight for height Z < -2) and overweight or obesity (weight for height Z>2) indicators were not analyzed since the number of outcome events was very low within subgroup categories.

[b] Height for age Z < -2

[c] Weight for age Z < -2

Table ST6. Average treatment effects on the treated for comparing children whose mothers had clinically significant depressive symptomatology with their estimated counterfactual of no maternal clinically significant depressive symptomatology

	Sex		Household Indigenous status		
	Males	Females	Non-Indigenous	Indigenous without Indigenous language spoken	Indigenous with Indigenous language spoken
	ATT MD (95% CI)				
**Nutritional status**					
Stunting[a]	4.6 (-1.7, 10.9)	7.6* (0.1, 15.0)	8.5* (0.6, 16.3)	9.5 (-1.4, 20.3)	-4.2 (-14.5, 6.0)
Underweight[b]	1.5 (-3.9, 6.9)	2.6 (-1.8, 7.0)	6.3* (0.4, 12.3)	-1.9 (-9.0, 5.3)	-0.3 (-5.2, 4.7)
**Child Development Evaluation Test 2nd Edition – EDI-II**					
Normal neurodevelopment	-12.5** (-21.5, -3.6)	6.7 (-5.5, 18.9)	0.7 (-11.8, 13.3)	-7.6 (-26.3, 11.0)	-4.8 (-12.6, 3.1)
**Bayley Scales of Infant and Toddler Development 3rd edition (BSID-III) composite scores**					
Cognitive	-1.25 (-3.66, 1.17)	0.52 (-1.78, 2.81)	1.68 (-3.22, 6.58)	-0.07 (-2.89, 2.74)	-2.34 (-4.99, 0.30)
Language	-2.30 (-6.03, 1.43)	-1.47 (-4.39, 1.44)	-1.60 (-7.44, 4.25)	-0.86 (-4.67, 2.95)	-3.57* (-6.61, -0.52)
Motor	-0.88 (-4.70, 2.94)	2.00 (-1.23, 5.24)	1.39 (-6.80, 9.58)	2.10 (-2.73, 6.92)	-1.46 (-3.65, 0.73)
Socio-emotional	-2.69 (-6.87, 1.49)	-2.48 (-5.20, 0.24)	-4.49 (-9.48, 0.51)	-2.51 (-6.65, 1.62)	-2.19 (-5.84, 1.47)
**Bayley Scales of Infant and Toddler Development 3rd edition (BSID-III) subtest scaled scores**					
Receptive language	-0.46 (-1.16, 0.25)	-0.22 (-0.86, 0.43)	-0.04 (-1.02, 0.95)	-0.23 (-0.97, 0.52)	-0.60* (-1.19, -0.01)
Expressive language	-0.30 (-0.95, 0.36)	-0.30 (-0.75, 0.16)	-0.51 (-1.68, 0.67)	-0.07 (-0.69, 0.56)	-0.61* (-1.16, -0.05)
Fine motor	-0.34 (-1.06, 0.39)	0.48 (-0.09, 1.05)	0.28 (-1.60, 2.15)	0.16 (-0.73, 1.04)	-0.13 (-0.84, 0.57)
Gross motor	0.05 (-0.72, 0.81)	0.18 (-0.50, 0.86)	0.22 (-0.77, 1.21)	0.52 (-0.28, 1.33)	-0.36 (-0.74, 0.02)
**Impaired neurodevelopment (BSID-III composite scores <85)**					
Cognitive	9.1 (-0.7, 18.9)	-4.5 (-12.9, 3.9)	-2.4 (-15.5, 10.8)	-2.9 (-13.2, 7.4)	6.3 (-2.1, 14.6)
Language	1.0 (-11.6, 13.6)	0.5 (-10.9, 12.0)	7.0 (-12.0, 26.0)	-4.2 (-15.9, 7.5)	3.5 (-8.0, 14.9)
Motor	6.1 (-6.1, 18.3)	-6.1 (-14.9, 2.6)	-4.7 (-25.2, 15.7)	-4.0 (-16.5, 8.4)	4.5 (-4.2, 13.2)

*p<0.05, **p<0.01, ***p<0.001
Maternal clinically significant depressive symptomatology was defined as a Center for Epidemiologic Studies Depression scale (CES-D) score of 16 or higher,
ATT MD: Average treatment effect on the treated as a mean difference, estimated with augmented inverse probability weighting; CI: Confidence interval.
Predictors for the outcomes and maternal depressive symptomatology status were sex, age in months, whether the child lives with a young sibling (<5 years), mother characteristics (age group, health insurance, educational status, occupational status, marital status and parity), and household characteristics (wealth index category, Indigenous status, affiliation to social programs and reported category of monthly income from all sources). For the analysis by household indigenous status, maternal age and schooling were specified as continuous since maternal symptomatology status did not vary in some categories of these characteristics.
Standard errors were adjusted for data dependencies within communities.
[a] Height for age Z < -2
[b] Weight for age Z < -2

Table ST7. Multiple logistic regressions for child nutritional status and the EDI-II neurodevelopment normal result among 1060 children aged 0 to 38 months from 24 communities in Oaxaca Mexico

	Stunting[a]		Low weight for age[b]		Overweight or Obese[c]		Normal development (EDI-II)[d]	
	AOR ± SE	P	AOR ± SE	P	AOR ± SE	P	AOR ± SE	P
Maternal depressive symptomatology								
CES-D≥16 vs CES-D<16	1.32 ± 0.16	0.025	1.28 ± 0.27	0.241	0.48 ± 0.18	0.046	0.86 ± 0.13	0.307
Sex								
Male	1 Ref.		1 Ref.		1 Ref.		1 Ref.	
Female	0.71 ± 0.09	0.009	0.82 ± 0.21	0.431	0.82 ± 0.38	0.661	1.42 ± 0.14	0.001
Age – mean age, months	1.00 ± 0.01	0.800	1.01 ± 0.02	0.349	0.95 ± 0.02	0.009	1.03 ± 0.01	<0.001
Young siblings								
None	1 Ref.		1 Ref.		1 Ref.		1 Ref.	
Has a sibling <5 years of age	1.63 ± 0.29	0.006	1.30 ± 0.56	0.536	1.02 ± 0.55	0.963	0.83 ± 0.15	0.305
Maternal age, years								
15 to 19	1 Ref.		1 Ref.		1 Ref.		1 Ref.	
20 to 29	0.74 ± 0.24	0.341	0.65 ± 0.27	0.300	1.44 ± 0.75	0.489	1.14 ± 0.34	0.668
30 to 49	0.76 ± 0.25	0.408	0.95 ± 0.31	0.887	0.89 ± 0.59	0.861	1.19 ± 0.43	0.639
Maternal completed education level								
None or elementary	1 Ref.		1 Ref.		1 Ref.		1 Ref.	
Middle school	0.93 ± 0.17	0.678	0.87 ± 0.26	0.654	1.53 ± 0.62	0.291	0.99 ± 0.16	0.968
Highschool	0.79 ± 0.13	0.147	0.64 ± 0.29	0.326	2.15 ± 0.83	0.048	0.98 ± 0.25	0.927
College/University	0.53 ± 0.16	0.038	0.36 ± 0.32	0.257	3.48 ± 1.94	0.026	1.25 ± 0.47	0.559
Health insurance								
None	1 Ref.		1 Ref.		1 Ref.		1 Ref.	
Seguro Popular	0.65 ± 0.13	0.034	1.53 ± 0.89	0.469	0.49 ± 0.30	0.245	0.92 ± 0.27	0.769
Social security	0.66 ± 0.20	0.166	0.37 ± 0.51	0.467	0.27 ± 0.24	0.138	0.73 ± 0.18	0.208
Maternal occupational status								
Has a job	1 Ref.		1 Ref.		1 Ref.		1 Ref.	
Housework	1.28 ± 0.30	0.295	1.37 ± 0.86	0.620	2.09 ± 2.00	0.440	1.46 ± 0.32	0.083
Housework with extra activities[e]	1.71 ± 0.39	0.020	2.20 ± 1.17	0.136	0.84 ± 0.94	0.875	1.30 ± 0.32	0.278
Self-employed	1.23 ± 0.28	0.375	1.96 ± 1.00	0.189	2.21 ± 1.73	0.312	0.94 ± 0.23	0.800
Other[f]	0.40 ± 0.21	0.088	2.68 ± 1.80	0.144	3.72 ± 4.05	0.227	0.39 ± 0.24	0.120
Marital status								

Single	1 Ref.		1 Ref.		1 Ref.		1 Ref.	
Free Union	0.54 ± 0.12	0.007	0.86 ± 0.40	0.741	1.10 ± 0.72	0.886	1.54 ± 0.36	0.064
Married	0.55 ± 0.12	0.005	0.79 ± 0.37	0.619	0.72 ± 0.56	0.666	1.71 ± 0.44	0.039
Separated/Divorced/Widowed	0.68 ± 0.23	0.255	0.53 ± 0.45	0.454	0.71 ± 0.39	0.534	1.61 ± 0.53	0.150
Parity-1	1.08 ± 0.07	0.273	0.95 ± 0.14	0.745	0.89 ± 0.31	0.729	0.87 ± 0.07	0.081
Household covariates								
Wealth index category								
Low	1 Ref.		1 Ref.		1 Ref.		1 Ref.	
Medium	0.68 ± 0.14	0.070	0.83 ± 0.28	0.585	1.04 ± 0.40	0.925	1.44 ± 0.26	0.041
High	0.57 ± 0.14	0.019	0.53 ± 0.26	0.196	0.84 ± 0.43	0.734	1.02 ± 0.19	0.904
Reported monthly income[g]								
Less than 189€	1 Ref.		1 Ref.		1 Ref.		1 Ref.	
189€ to 314€	0.80 ± 0.17	0.289	0.87 ± 0.29	0.667	1.04 ± 0.40	0.927	1.40 ± 0.24	0.052
>314€ to 440€	0.77 ± 0.17	0.229	0.44 ± 0.25	0.153	0.94 ± 0.49	0.907	1.54 ± 0.32	0.036
More than 440€	0.43 ± 0.16	0.022	0.26 ± 0.21	0.104	3.38 ± 2.07	0.046	2.19 ± 0.57	0.003
Indigenous status								
Non-Indigenous	1 Ref.		1 Ref.		1 Ref.		1 Ref.	
Indigenous without Indigenous language spoken	0.91 ± 0.16	0.613	0.54 ± 0.13	0.008	1.30 ± 0.49	0.485	1.18 ± 0.19	0.284
Indigenous with Indigenous language spoken	0.92 ± 0.22	0.729	0.40 ± 0.18	0.046	0.29 ± 0.17	0.031	0.69 ± 0.12	0.036
Number of social programs								
None	1 Ref.		1 Ref.		1 Ref.		1 Ref.	
One	1.20 ± 0.26	0.402	0.85 ± 0.22	0.530	0.94 ± 0.42	0.890	0.96 ± 0.15	0.782
Two to four	0.85 ± 0.31	0.662	0.70 ± 0.44	0.566	1.20 ± 0.91	0.815	1.03 ± 0.39	0.941
Reference odds[h]	1.30 ± 0.63	0.586	0.14 ± 0.09	0.003	0.02 ± 0.02	0.001	0.29 ± 0.15	0.018

AOR: adjusted odds ratio; SE: standard error; CES-D: Center for Epidemiologic Studies depression scale.
Standard errors were adjusted for data dependencies within communities.
[a]Height for age Z < -2. The term "height" refers to length (children aged < 24months) or stature (children aged ≥24 months)
[b]Weight for age Z < -2.[c]Weight for height Z > 2
[d]Normal test result from the Child Development Evaluation Test 2nd Edition (*Evaluación del Desarrollo Infantil,* in Spanish) - EDI II.
[e]Extra activities such as helping in a business or selling a product.
[f]Includes being a student or retired.
[g]Converted from Mexican pesos to euros using the 2020 purchase power parity exchange rates for private consumption.
[h]Odds of the outcome when all variables are set at their reference category, child's age at the overall mean (19.6 months) and parity at 1.

Table ST8. Multiple linear regression models for Bayley Scales of Infant and Toddler Development 3rd edition composite scores in a subsample of 554 children from 24 communities in Oaxaca Mexico

	Cognitive score		Language score		Motor score		Socioemotional score	
	AMD ± SE	P	AMD ± SE	P	AMD ± SE	P	AMD ± SE	P
Maternal depressive symptomatology								
CES-D≥16 vs CES-D<16	-0.29 ± 0.87	0.741	-1.43 ± 1.31	0.288	0.80 ± 1.23	0.522	-2.31 ± 1.04	0.039
Sex								
Male				Reference				
Female	0.77 ± 0.88	0.390	2.26 ± 0.73	0.005	1.83 ± 0.87	0.047	0.65 ± 0.77	0.408
Age – mean age, months	-0.19 ± 0.06	0.007	-0.08 ± 0.07	0.307	0.33 ± 0.08	0.001	-0.20 ± 0.06	0.004
Young siblings								
None				Reference				
Has a sibling <5 years of age	-2.37 ± 0.91	0.016	-3.21 ± 1.23	0.016	-0.31 ± 1.33	0.819	-1.81 ± 1.97	0.369
Maternal age, years								
15 to 19				Reference				
20 to 29	-0.89 ± 1.32	0.508	-0.02 ± 1.70	0.993	-2.43 ± 1.92	0.220	-5.15 ± 2.50	0.051
30 to 49	-1.10 ± 1.43	0.453	-0.53 ± 1.99	0.792	-2.52 ± 2.23	0.270	-2.97 ± 2.55	0.257
Maternal completed education level								
None or elementary				Reference				
Middle school	2.37 ± 1.11	0.045	2.13 ± 0.95	0.035	2.09 ± 1.11	0.073	0.13 ± 1.67	0.938
Highschool	2.80 ± 1.39	0.057	2.36 ± 1.16	0.055	-0.30 ± 1.74	0.864	2.98 ± 1.76	0.105
College/University	6.57 ± 1.78	0.001	4.94 ± 1.69	0.008	4.05 ± 2.45	0.112	1.98 ± 2.19	0.378
Health insurance								
None				Reference				
Seguro Popular	-2.29 ± 1.04	0.038	-0.20 ± 2.17	0.929	1.11 ± 1.90	0.566	2.15 ± 1.73	0.228
Social security	-1.75 ± 1.45	0.243	-2.59 ± 2.53	0.318	2.43 ± 2.73	0.384	0.14 ± 2.46	0.956
Maternal occupational status								
Has a job				Reference				
Housework	1.84 ± 1.38	0.194	0.54 ± 1.20	0.656	1.96 ± 2.16	0.373	-1.91 ± 1.41	0.189
Housework with extra activities[a]	3.04 ± 1.35	0.035	0.36 ± 1.70	0.836	1.29 ± 2.24	0.571	-1.34 ± 1.32	0.322

Self-employed	0.15 ± 1.21	0.904	-0.54 ± 1.54	0.730	1.30 ± 1.81	0.482	-1.16 ± 1.40	0.417
Other[b]	-1.45 ± 3.56	0.688	-5.04 ± 3.17	0.126	-3.33 ± 3.78	0.388	-5.14 ± 3.05	0.107
Marital status								
Single				Reference				
Free Union	0.21 ± 1.68	0.903	3.76 ± 1.29	0.008	0.70 ± 2.05	0.737	-2.54 ± 1.43	0.090
Married	1.87 ± 2.38	0.442	3.42 ± 1.28	0.014	1.77 ± 2.66	0.512	-2.27 ± 1.86	0.236
Separated/Divorced/Widowed	4.48 ± 1.67	0.014	5.52 ± 3.47	0.127	6.42 ± 4.53	0.170	-0.75 ± 2.50	0.767
Parity-1	-0.34 ± 0.47	0.481	0.46 ± 0.52	0.383	-0.39 ± 0.61	0.523	-0.19 ± 0.69	0.791
Household covariates								
Wealth index category								
Low				Reference				
Medium	1.73 ± 1.06	0.117	0.36 ± 1.11	0.749	2.67 ± 1.59	0.108	1.90 ± 1.69	0.273
High	1.54 ± 1.02	0.144	2.53 ± 1.37	0.079	2.72 ± 1.51	0.086	4.62 ± 2.13	0.042
Reported monthly income[c]								
Less than 189€				Reference				
189€ to 314€	1.09 ± 1.19	0.369	1.00 ± 1.24	0.427	2.63 ± 1.25	0.048	-1.21 ± 1.36	0.384
>314€ to 440€	1.05 ± 1.77	0.559	2.43 ± 1.40	0.097	4.10 ± 2.12	0.067	0.84 ± 1.39	0.557
More than 440€	0.89 ± 1.37	0.526	3.33 ± 1.84	0.085	2.73 ± 1.68	0.122	3.81 ± 2.20	0.101
Indigenous status								
Non-Indigenous				Reference				
Indigenous without Indigenous language spoken	-0.22 ± 1.18	0.856	0.34 ± 0.85	0.694	-0.04 ± 1.36	0.978	2.52 ± 1.33	0.072
Indigenous with Indigenous language spoken	-1.68 ± 0.98	0.103	-1.46 ± 1.55	0.358	-1.64 ± 1.08	0.142	4.41 ± 1.48	0.007
Number of social programs								
None				Reference				
One	1.51 ± 1.00	0.148	-0.08 ± 0.91	0.928	-1.32 ± 0.95	0.180	-0.53 ± 1.51	0.729
Two to four	-2.26 ± 1.91	0.251	2.74 ± 2.30	0.246	0.96 ± 1.83	0.606	1.27 ± 4.05	0.757
Reference mean[d]	92.5 ± 2.2		85.0 ± 2.9		88.9 ± 3.8		91.9 ± 3.7	

AMD: adjusted mean difference; SE: standard error; CES-D: Center for Epidemiologic Studies depression scale.
Standard errors were adjusted for data dependencies within communities.
[a] Extra activities such as helping in a business or selling a product.
[b] Includes being student or retired.
[c] Converted from Mexican pesos to euros using the 2020 purchase power parity exchange rates for private consumption.
[d] Mean of the outcome when all variables are set at their reference category, child's age at the overall mean (19.6 months) and parity at 1.

Table ST9. Multiple linear regression models for Bayley Scales of Infant and Toddler Development 3rd edition subtest scaled scores in a subsample of 554 children from 23 communities in Oaxaca Mexico

	Receptive language		Expressive language		Fine motor		Gross motor	
	AMD ± SE	P	AMD ± SE	P	AMD ± SE	P	AMD ± SE	P
Maternal depressive symptomatology								
CES-D≥16 vs CES-D<16	-0.26 ± 0.23	0.270	-0.22 ± 0.24	0.353	0.08 ± 0.24	0.740	0.19 ± 0.23	0.424
Sex								
Male	Reference							
Female	0.47 ± 0.17	0.010	0.31 ± 0.13	0.033	0.49 ± 0.18	0.013	0.11 ± 0.15	0.494
Age – mean age, months	0.04 ± 0.01	0.017	-0.06 ± 0.01	<0.001	0.04 ± 0.02	0.012	0.07 ± 0.02	0.001
Young siblings								
None	Reference							
Has a sibling <5 years of age	-0.59 ± 0.22	0.016	-0.51 ± 0.21	0.026	-0.13 ± 0.22	0.543	0.03 ± 0.29	0.904
Maternal age, years								
15 to 19	Reference							
20 to 29	-0.07 ± 0.44	0.872	0.09 ± 0.25	0.707	-0.49 ± 0.41	0.245	-0.32 ± 0.41	0.442
30 to 49	-0.10 ± 0.51	0.848	-0.04 ± 0.29	0.888	-0.43 ± 0.41	0.297	-0.41 ± 0.44	0.360
Maternal completed education level								
None or elementary	Reference							
Middle school	0.48 ± 0.21	0.030	0.26 ± 0.14	0.089	0.41 ± 0.18	0.032	0.27 ± 0.25	0.289
Highschool	0.45 ± 0.25	0.083	0.36 ± 0.21	0.107	0.08 ± 0.30	0.798	-0.18 ± 0.32	0.570
College/University	1.05 ± 0.31	0.003	0.64 ± 0.33	0.065	0.91 ± 0.58	0.134	0.44 ± 0.51	0.396
Health insurance								
None	Reference							
Seguro Popular	0.13 ± 0.40	0.755	-0.20 ± 0.41	0.625	0.00 ± 0.34	0.995	0.39 ± 0.36	0.285
Social security	-0.28 ± 0.43	0.522	-0.62 ± 0.51	0.239	0.51 ± 0.44	0.257	0.30 ± 0.57	0.601
Maternal occupational status								
Has a job	Reference							
Housework	0.23 ± 0.24	0.343	-0.04 ± 0.22	0.873	0.53 ± 0.31	0.103	0.12 ± 0.47	0.805
Housework with extra activities[a]	0.36 ± 0.35	0.309	-0.22 ± 0.29	0.452	0.46 ± 0.33	0.176	-0.04 ± 0.45	0.937
Self-employed	-0.03 ± 0.28	0.918	-0.15 ± 0.29	0.604	0.26 ± 0.32	0.433	0.18 ± 0.39	0.651
Other[b]	-0.84 ± 0.55	0.142	-0.87 ± 0.61	0.164	-0.62 ± 0.76	0.423	-0.47 ± 0.64	0.467

Marital status								
Single				Reference				
Free Union	0.58 ± 0.27	0.043	0.74 ± 0.29	0.017	0.05 ± 0.44	0.914	0.20 ± 0.35	0.577
Married	0.60 ± 0.24	0.018	0.60 ± 0.33	0.082	0.19 ± 0.53	0.725	0.40 ± 0.43	0.361
Separated/Divorced/Widowed	1.16 ± 0.69	0.109	0.74 ± 0.55	0.198	0.88 ± 0.86	0.316	1.26 ± 0.82	0.137
Parity-1	0.10 ± 0.11	0.342	0.04 ± 0.09	0.659	0.04 ± 0.11	0.704	-0.17 ± 0.12	0.169
Household covariates								
Wealth index category								
Low				Reference				
Medium	-0.17 ± 0.20	0.408	0.28 ± 0.21	0.194	0.30 ± 0.30	0.328	0.60 ± 0.30	0.058
High	0.43 ± 0.25	0.100	0.42 ± 0.25	0.116	0.34 ± 0.25	0.197	0.58 ± 0.31	0.079
Reported monthly income[c]								
Less than 189€				Reference				
189€ to 314€	0.11 ± 0.24	0.642	0.26 ± 0.27	0.338	0.65 ± 0.24	0.014	0.22 ± 0.22	0.338
>314€ to 440€	0.14 ± 0.27	0.612	0.71 ± 0.33	0.044	0.58 ± 0.43	0.192	0.76 ± 0.33	0.030
More than 440€	0.74 ± 0.41	0.087	0.39 ± 0.31	0.215	0.37 ± 0.32	0.273	0.55 ± 0.34	0.118
Indigenous status								
Non-Indigenous				Reference				
Indigenous without Indigenous language spoken	0.07 ± 0.21	0.732	0.04 ± 0.14	0.783	-0.10 ± 0.23	0.679	0.09 ± 0.27	0.751
Indigenous with Indigenous language spoken	-0.07 ± 0.34	0.834	-0.45 ± 0.23	0.069	-0.26 ± 0.23	0.268	-0.27 ± 0.15	0.084
Number of social programs								
None				Reference				
One	0.00 ± 0.22	1.000	-0.01 ± 0.15	0.947	-0.12 ± 0.28	0.674	-0.33 ± 0.13	0.020
Two to four	0.37 ± 0.50	0.468	0.55 ± 0.36	0.137	0.17 ± 0.45	0.703	0.13 ± 0.24	0.602
Reference mean[d]	7.22 ± 0.73		7.53 ± 0.43		8.71 ± 0.70		7.53 ± 0.69	

AMD: adjusted mean difference; SE: standard error; CES-D: Center for Epidemiologic Studies depression scale; SD: standard deviation.
Standard errors were adjusted for data dependencies within communities.
[a] Extra activities such as helping in a business or selling a product.
[b] Includes being student or retired.
[c] Converted from Mexican pesos to euros using the 2020 purchase power parity exchange rates for private consumption.
[d] Mean of the outcome when all variables are set at their reference category, child's age at the overall mean (19.6 months) and parity at 1.

Table ST10. Covariate-adjusted associations between clinically significant depressive symptomatology and child development outcomes from complete case analyses

Outcome	Total	Sex		Household Indigenous status		
		Males	Females	Non-Indigenous	Indigenous without Indigenous language spoken	Indigenous with Indigenous language spoken
**Nutritional status indicators, adjusted odds ratio (95% CI)**						
Stunting[a]	1.37* (1.06, 1.77)	1.23 (0.88, 1.71)	1.55 (0.99, 2.41)	1.61** (1.15, 2.24)	1.47 (0.90, 2.38)	0.97 (0.61, 1.54)
Underweight[b]	1.23 (0.79, 1.92)	1.08 (0.53, 2.21)	1.47 (0.68, 3.16)	2.68** (1.31, 5.52)	0.52 (0.21, 1.31)	0.53 (0.13, 2.16)
Overweight[c]	0.44* (0.23, 0.85)					
Normal neurodevelopment	0.78 (0.58, 1.07)	0.51*** (0.34, 0.74)	1.18 (0.66, 2.13)	0.85 (0.46, 1.56)	0.68 (0.34, 1.35)	0.90 (0.69, 1.18)
**Bayley-III composite scales of development, adjusted mean difference (95% CI)**						
Cognitive	-0.65 (-2.70, 1.40)	-1.10 (-3.69, 1.50)	-0.27 (-3.31, 2.76)	0.57 (-2.71, 3.85)	0.07 (-2.83, 2.97)	-3.02* (-5.66, -0.39)
Language	-1.82 (-5.04, 1.40)	-1.55 (-5.32, 2.21)	-2.05 (-5.81, 1.72)	-2.16 (-9.05, 4.74)	0.21 (-3.28, 3.69)	-4.74*** (-7.10, -2.39)
Motor	-0.03 (-3.05, 2.98)	-2.09 (-5.28, 1.11)	1.69 (-2.83, 6.21)	-0.26 (-6.47, 5.94)	1.82 (-2.20, 5.84)	-2.78 (-6.75, 1.19)
Socioemotional	-1.59 (-4.27, 1.10)	-0.95 (-4.57, 2.68)	-2.12 (-5.55, 1.31)	-2.13 (-6.96, 2.70)	-1.95 (-5.04, 1.15)	-0.45 (-4.32, 3.41)
**Bayley-III subtest scaled scores, adjusted mean difference (95% CI)**						
Receptive language	-0.41 (-0.99, 0.17)	-0.40 (-1.15, 0.35)	-0.41 (-1.07, 0.25)	-0.17 (-1.32, 0.98)	-0.22 (-0.91, 0.48)	-0.95** (-1.61, -0.29)
Expressive language	-0.21 (-0.77, 0.34)	-0.10 (-0.72, 0.53)	-0.31 (-1.01, 0.39)	-0.56 (-1.96, 0.84)	0.30 (-0.29, 0.88)	-0.68*** (-0.96, -0.40)
Fine motor	-0.10 (-0.68, 0.49)	-0.52 (-1.17, 0.14)	0.25 (-0.58, 1.09)	-0.02 (-1.57, 1.53)	0.18 (-0.55, 0.91)	-0.62 (-1.34, 0.09)
Gross motor	0.08 (-0.42, 0.58)	-0.17 (-0.75, 0.40)	0.30 (-0.46, 1.06)	-0.05 (-0.75, 0.65)	0.41 (-0.25, 1.06)	-0.31 (-1.22, 0.60)
**Bayley-III impaired[f] development indicators, adjusted odds ratio (95% CI)**						
Cognitive	1.13 (0.57, 2.22)	1.75 (0.62, 4.96)	0.80 (0.36, 1.75)	1.33 (0.49, 3.61)	0.66 (0.17, 2.56)	1.78 (0.82, 3.86)
Language	1.10 (0.65, 1.87)	1.08 (0.57, 2.06)	1.12 (0.48, 2.61)	1.18 (0.40, 3.44)	0.94 (0.47, 1.84)	1.30 (0.72, 2.35)
Motor	1.13 (0.61, 2.10)	2.24* (1.04, 4.78)	0.61 (0.25, 1.47)	1.47 (0.54, 4.00)	0.65 (0.25, 1.73)	1.92 (0.80, 4.57)

*p<0.05, **p<0.01, ***p<0.001. CES-D: Center for Epidemiologic Studies depression scale. Adjustment variables were sex, age in months, whether the child lives with a young sibling (<5 years), mother characteristics (age group, health insurance, educational status, occupational status, marital status and parity), and household characteristics (wealth index category, Indigenous status, affiliation to social programs and reported category of monthly income from all sources). Standard errors were adjusted for data dependencies within communities; [a] Height for age Z < -2; [b] Weight for age Z < -2; [c] Overweight or obesity (weight for height Z>2) was not analyzed by subgroups since the number of outcome events was very low within subgroups.

Table ST11. Covariate-adjusted associations between CES-D standardized scores and child development outcomes from complete case analyses

Outcome	Total	Sex		Household Indigenous status		
		Males	Females	Non-Indigenous	Indigenous without Indigenous language spoken	Indigenous with Indigenous language spoken
**Nutritional status indicators, adjusted odds ratio (95% CI)**						
Stunting[a]	1.12* (1.01, 1.23)	1.01 (0.87, 1.17)	1.26* (1.05, 1.52)	1.09 (0.96, 1.23)	1.27* (1.02, 1.57)	0.94 (0.75, 1.17)
Underweight[b]	1.13 (0.94, 1.35)	1.11 (0.87, 1.42)	1.15 (0.92, 1.44)	1.34** (1.08, 1.68)	0.94 (0.73, 1.20)	0.91 (0.52, 1.61)
Overweight[c]	0.63** (0.44, 0.89)					
Normal neurodevelopment	0.90 (0.78, 1.03)	0.79** (0.68, 0.93)	1.02 (0.76, 1.39)	1.02 (0.85, 1.23)	0.85 (0.64, 1.11)	0.79** (0.67, 0.94)
**BSID-III composite scales of development, adjusted mean difference (95% CI)**						
Cognitive	-0.34 (-1.13, 0.46)	-0.39 (-1.65, 0.86)	-0.28 (-1.64, 1.07)	0.42 (-0.93, 1.77)	-0.20 (-1.41, 1.01)	-1.61* (-2.87, -0.35)
Language	-0.58 (-1.98, 0.82)	-0.57 (-2.10, 0.97)	-0.59 (-2.33, 1.15)	-0.20 (-2.18, 1.78)	-0.03 (-1.84, 1.78)	-2.19*** (-2.98, -1.41)
Motor	0.28 (-1.02, 1.58)	0.20 (-1.34, 1.73)	0.36 (-1.55, 2.27)	1.10 (-0.69, 2.88)	0.57 (-1.32, 2.45)	-1.38 (-3.36, 0.60)
Socioemotional	-0.77 (-2.05, 0.51)	-0.84 (-2.60, 0.91)	-0.69 (-2.32, 0.93)	-0.10 (-2.44, 2.23)	-1.28 (-3.07, 0.51)	-0.61 (-2.39, 1.18)
**BSID-III subtest scaled scores, adjusted mean difference (95% CI)**						
Receptive language	-0.15 (-0.42, 0.11)	-0.16 (-0.46, 0.13)	-0.14 (-0.47, 0.19)	0.04 (-0.34, 0.42)	-0.13 (-0.47, 0.22)	-0.45*** (-0.67, -0.23)
Expressive language	-0.04 (-0.29, 0.20)	-0.02 (-0.29, 0.26)	-0.07 (-0.38, 0.24)	-0.09 (-0.50, 0.31)	0.12 (-0.21, 0.44)	-0.30*** (-0.46, -0.14)
Fine motor	-0.01 (-0.26, 0.25)	-0.10 (-0.38, 0.18)	0.08 (-0.30, 0.45)	0.12 (-0.24, 0.47)	0.04 (-0.36, 0.45)	-0.28 (-0.76, 0.21)
Gross motor	0.10 (-0.10, 0.30)	0.17 (-0.13, 0.46)	0.03 (-0.27, 0.34)	0.25 (-0.06, 0.55)	0.14 (-0.10, 0.38)	-0.18 (-0.48, 0.12)
**Impaired development (BSID-III composite scores <85), adjusted odds ratio (95% CI)**						
Cognitive	1.07 (0.81, 1.40)	1.21 (0.83, 1.75)	0.96 (0.65, 1.41)	1.09 (0.74, 1.59)	0.68 (0.39, 1.21)	1.65* (1.12, 2.42)
Language	1.05 (0.82, 1.33)	1.01 (0.72, 1.40)	1.09 (0.76, 1.58)	0.99 (0.75, 1.31)	1.00 (0.66, 1.51)	1.23 (0.97, 1.57)
Motor	0.97 (0.69, 1.37)	1.08 (0.68, 1.73)	0.89 (0.63, 1.24)	0.94 (0.56, 1.59)	0.86 (0.51, 1.42)	1.21 (0.85, 1.72)

*p<0.05, **p<0.01, ***p<0.001. CES-D: Center for Epidemiologic Studies depression scale. BSID-III: Bayley Scales of Infant and Toddler Development 3rd edition. Adjustment variables were sex, age in months, whether the child lives with a young sibling (<5 years), mother characteristics (age group, health insurance, educational status, occupational status, marital status and parity), and household characteristics (wealth index category, Indigenous status, affiliation to social programs and reported category of monthly income from all sources). Standard errors were adjusted for data dependencies within communities; [a] Height for age Z < -2; [b] Weight for age Z < -2; [c] Overweight or obesity (weight for height Z>2) was not analyzed by subgroups since the number of outcome events was very low within subgroups.

Table ST12. Covariate-adjusted associations between the transformed scale of maternal depressive symptomatology and nutritional status and neurodevelopment outcomes among children aged 0 to 38 months from 24 communities in Oaxaca Mexico

	CES-D score transformations	
	$ZCESD = (\text{CES-D} - 12.7)/10.0$	$(\text{CES-D}^{0.332})/0.662$
Non-parametric skewness[a]	0.27[b]	0.00
**Covariate-adjusted[c] associations with nutritional status indicators, odds ratio[d] (95% CI)**		
Stunting	1.09 (0.97, 1.22)	1.13* (1.00, 1.27)
Underweight	1.09 (0.90, 1.32)	1.18 (0.93, 1.50)
Overweight	0.69 (0.47, 1.01)	0.69** (0.54, 0.89)
Normal neurodevelopment	0.94 (0.82, 1.07)	0.97 (0.85, 1.12)
**Covariate-adjusted[c] associations with Bayley-III composite scales of development, Mean difference[e] (95% CI)**		
Cognitive	-0.10 (-0.80, 0.60)	0.07 (-0.75, 0.88)
Language	-0.39 (-1.62, 0.84)	-0.36 (-1.60, 0.89)
Motor	0.58 (-0.48, 1.65)	0.76 (-0.39, 1.91)
Socioemotional	-1.20 (-2.44, 0.04)	-1.16 (-2.48, 0.17)
**Covariate-adjusted[c] associations with Bayley-III subtest scaled scores, Mean difference[e] (95% CI)**		
Receptive language	-0.07 (-0.32, 0.17)	-0.09 (-0.32, 0.15)
Expressive language	-0.06 (-0.27, 0.16)	-0.03 (-0.25, 0.20)
Fine motor	0.06 (-0.15, 0.26)	0.07 (-0.14, 0.28)
Gross motor	0.14 (-0.05, 0.32)	0.18 (-0.02, 0.39)
**Covariate-adjusted[c] associations with Bayley-III impaired[f] development indicators, Odds ratio[d] (95% CI)**		
Cognitive	1.05 (0.79, 1.39)	1.06 (0.75, 1.49)
Language	0.99 (0.80, 1.23)	0.96 (0.77, 1.20)
Motor	0.90 (0.67, 1.20)	0.90 (0.66, 1.22)

95% Confidence intervals are shown in parentheses.
*p<0.05, **p<0.01, ***p<0.001
CES-D: Center for Epidemiologic Studies depression scale
[a] Defined as the difference between the mean and median in standard deviation units.
[b] It also corresponds to the skewness of the untransformed CES-D scores.
[c] Adjustment variables included sex, age in months, whether the child lives with a young sibling (<5 years), mother characteristics (age group, health insurance, educational status, occupational status, marital status and parity), and household characteristics (wealth index category, Indigenous status, affiliation to social programs and reported category of monthly income from all sources). Standard errors were adjusted for data dependencies within communities.
[d] Odds ratio comparing the odds of the outcome between CES-D transformed scores that differ by one standard deviation.
[e] Mean difference corresponding to an associated increase of one unit in the transformed CES-D score.
[f] Mild, moderate or severe impairment defined as a composite score < 85
